# PD-1 Blockade Restores the Proliferation of Peripheral Blood Lymphocyte and Inhibits Lymphocyte Apoptosis in a BALB/c Mouse Model of CP BVDV Acute Infection

**DOI:** 10.3389/fimmu.2021.727254

**Published:** 2021-09-06

**Authors:** Yu Liu, Chenhua Wu, Nannan Chen, Yang Li, Chunling Fan, Shangqi Zhao, Tongtong Bai, Zhibo Zhao, Jinwei Chen, Siyu Su, Zecai Zhang, Yulong Zhou, Zhanbo Zhu

**Affiliations:** ^1^College of Animal Science and Veterinary Medicine, HeiLongJiang BaYi Agricultural University, Daqing, China; ^2^Heilongjiang Provincial Engineering Research Center for Prevention and Control of Cattle Diseases, HeiLongJiang BaYi Agricultural University, Daqing, China; ^3^Heilongjiang Provincial Key Laboratory of Prevention and Control of Bovine Diseases, HeiLongJiang BaYi Agricultural University, Daqing, China; ^4^College of Engineering, HeiLongJiang BaYi Agricultural University, Daqing, China; ^5^Heilongjiang Province Cultivating Collaborative Innovation Center for The Beidahuang Modern Agricultural Industry Technology, HeiLongJiang BaYi Agricultural University, Daqing, China

**Keywords:** programmed death-1, bovine viral diarrhea virus, immune dysfunction, lymphopenia, mouse model

## Abstract

Acute infection of bovine viral diarrhea virus (BVDV) is associated with immune dysfunction and can cause peripheral blood lymphopenia and lymphocyte apoptosis. Our previous study has confirmed that programmed death-1 (PD-1) blockade inhibits peripheral blood lymphocytes (PBL) apoptosis and restores proliferation and anti-viral immune functions of lymphocytes after BVDV infection *in vitro*. However, the situation *in vivo* remains to be further studied and confirmed. Therefore, in this study, we established a BALB/c mouse model of acute BVDV infection with cytopathic (CP) BVDV (strain NADL) and non-cytopathic (NCP) BVDV (strain NY-1). Then, we examined the mRNA and protein levels of PD-1 and programmed death-ligand 1 (PD-L1) in peripheral blood mononuclear cells (PBMC) from BVDV-infected mice and analyzed the effects of PD-1 blockade on the proportions of CD3^+^, CD4^+^, and CD8^+^ T cell subsets, the apoptosis and proliferation of PBL, and the production of IL-2 and IFN-γ. We found that leukopenia, lymphocytopenia, and thrombocytopenia were developed in both CP and NCP BVDV-infected mice at day 7 of post-infection. The mRNA and protein expression of PD-1 and PD-L1 were significantly upregulated in CP and NCP BVDV-infected mice. Moreover, PD-1/PD-L1 upregulation was accompanied by leukopenia and lymphopenia. Additionally, PD-1 blockade inhibited PBL apoptosis and virus replication, restored the proportions of CD3^+^, CD4^+^, and CD8^+^ T cell subsets, and increased IFN-γ production and p-ERK expression in BVDV-infected mice. However, blocking PD-1 did not significantly affect PBL proliferation and IL-2 production in NCP BVDV-infected mice. Our findings further confirmed the immunomodulatory role of PD-1 in peripheral blood lymphocytopenia *in vivo* and provided a scientific basis for exploring the molecular mechanism of immune dysfunction caused by acute BVDV infection.

## Introduction

Bovine viral diarrhea virus (BVDV) infection can lead to bovine viral diarrhea-mucosal disease (BVD-MD) and cause fever, mucosal erosions and necrosis, diarrhea, thrombocytopenia, peripheral blood lymphopenia, abortion, and severe congenital abnormalities (1). BVD-MD is a key quarantinable infectious disease in cattle farms and international trade with a widespread worldwide distribution, which has caused great losses to the cattle industry (2). BVDV belongs to the Flaviviridae family, genus Pestivirus (3). Flaviviridae also includes critical human pathogens (4, 5) such as hepatitis C virus (HCV), yellow fever virus (YFV), West Nile virus (WNV), Japanese encephalitis virus (JEV), Saint Louis encephalitis virus (SLEV), and Dengue virus (DV). BVDV is classified as two biotypes, cytopathic (CP) and non-cytopathic (NCP), based on the effect of the infection on cell culture (6).

Acute BVDV infection can lead to peripheral blood lymphopenia and apoptosis and is associated with immune dysfunction (7). Besides BVDV, SARS-CoV (8), MERS-CoV, SARS-CoV-2 (9), human immunodeficiency virus (HIV), and avian influenza virus (AIV) can also result in severe lymphocytopenia (10). Thus, the immunopathological mechanisms of lymphopenia caused by acute BVDV infection have been widely concerned and are of great significance for the prevention and control of BVDV.

The programmed death-1 (PD-1) pathway induces functional exhaustion of lymphocyte, inhibition of proliferation and apoptosis of lymphocyte during acute and chronic viral infections, such as HIV (11), HCV (12), and bovine leukemia virus (BLV) (13). More notably, blocking the PD-1 pathway by antibodies improves lymphocyte function (14, 15), inhibits viral replication (13), and decreases lymphocyte apoptosis (16). Our previous studies (17, 18) have found that PD-1 blockade inhibits peripheral blood lymphocyte (PBL) apoptosis and restores proliferation and anti-viral immune functions of PBL *in vitro*. Remarkably, the PD-1/PD-L1 interaction has a more substantial effect on the immunoregulation of inhibiting proliferation induced by CP BVDV infection. The immunomodulatory effects of the PD-1 pathway on PBL in acute infection of CP and NCP BVDV need to be further studied *in vivo*.

Previous research (19) on BVDV assessing the potential of using mice as a model for BVDV has shown that intraperitoneal (IP) injection could successfully induce BVDV infection in mice. To further clarify the role of PD-1 in acute BVDV infection *in vivo*, in this study, we established a BALB/c mouse model of acute BVDV infection by reference to a previous study (19). Then, we investigated the effects of PD-1 pathway on the apoptosis and proliferation of PBL by the PD-1 blockade in the mouse model of BVDV infection. Our findings provide a scientific basis for exploring the molecular mechanism of immune dysfunction caused by acute BVDV infection in the mouse model.

## Materials and Methods

### Ethics Statement

This study was carried out in accordance with the principles of the Basel Declaration and recommendations by the guidelines set from the College of Animal Science and Veterinary Medicine, HeiLongJiang BaYi Agricultural University. The protocol was approved by the Management Committee of the Experimental Animal Center (MCEAC) of Heilongjiang Bayi Agricultural University (MCEAC-2020-0018). All standard biosecurity and institutional safety procedures from MCEAC been adhered during the study period.

### BVDV Infection of Mice

Specific pathogen-free BALB/c mice (6-8 weeks old, 18-22g) were purchased from the Laboratory Animal Department of Harbin Medical University (Harbin, China). All animals were maintained under pathogen-free conditions and handled in strict accordance with the guidelines and protocols approved for these experiments by the Management Committee of the Experimental Animal Center of Heilongjiang Bayi Agricultural University.

The CP BVDV-1a (strain NADL, No. VR-534) and NCP BVDV-1b (strain NY-1, No. VR-524) were from the American Type Culture Collection (ATCC, Manassas, VA, USA). Fifteen mice were assigned into the CP BVDV-, NCP BVDV-, and mock-infection groups with 5 mice per group. Challenge was performed by intraperitoneal (IP) injection of 0.4 mL of culture medium containing 10^6^ copies/mL of each virus (20). Mock-infected mice were given 0.4 mL of DMEM (Gibco, Grand Island, NY, USA) *via* IP injection. At days 4, 7, and 10 of post-infection, all mice were sacrificed by cervical dislocation to collect blood, tissue, and feces samples as previously reported (20). The effects of BVDV infection were evaluated through clinical, hematological, virological, immunohistochemical (IHC), and histopathological assessments.

### Hematological Assessment

Blood samples with EDTA anticoagulant were used to perform the hematological analysis. White blood cells (WBC), lymphocytes (LYM), and platelets (PLT) were counted with the IDEXX VetAutoread hematology analyzer (IDEXX Laboratories, Inc., Westbrook, ME, USA) according to the manufacturer’s instructions.

### Detection of Virus Replication

To evaluate the virus replication in mouse tissues, feces, and blood, the nucleotide sequence of 5′ non-coding region (NCR) of BVDV was amplified and cloned into a pMD18-T vector (18). The recombinant plasmid pMD18-T/5′NCR was serially diluted 10-fold in TE buffer, pH 8.0, to make template standards with known copy numbers. Each dilution was tested in triplicate as the amplification template to construct standard curves to determine the copy number of the BVDV 5′NCR gene. Quantitative real-time PCR (qRT-PCR) was performed in the CFX96 Touch Real-Time PCR Detection System (Bio-Rad, Hercules, CA, USA) using SYBR Premix Ex Taq II (RR820A, TaKaRa Biotechnology, Dalian, China) following the manufacturer’s instructions. Primers used in the qRT-PCR were 5′-GAGTACAGGGT AGTCGTCAG-3′ and 5′-CTCTGCAGCACCCTATCAGG-3′ for 5′ NCR and 5′-CGC ACCACTGGCATTGTCAT-3′ and 5′-TCCAAGGCGACGTAGCAGAG-3′ for β-actin. Each cDNA sample from peripheral blood mononuclear cells (PBMC) suspension was tested in triplicate. The cycling conditions were initial template denaturing at 95°C for 30 s, followed by45 cycles of template amplification at 95°C for 5 s, 60°C for 30 s, and 72°C for 30 s. A final melting curve analysis was performed from 65°C to 95°C at a rate of 0.1°C/s (continuous acquisition), with a final cooling to 40°C. The results were shown as the mean copy number per gram of tissue or per milliliter of whole blood.

### Immunohistochemistry and Histopathology

Five mice from each BVDV strain or mock infection group were sacrificed by cervical dislocation and necropsied for harvesting tissues at day 7 of post-infection. For BVDV antigen detection, formalin-fixed paraffin-embedded blocks of mouse tissues, including the heart, liver, spleen, lung, colon, ileum, and duodenum, were subjected to immunohistochemical (IHC) staining analyses. Briefly, 4-6 μm-thick paraffin-embedded tissue sections were deparaffinized in xylene, rehydrated through a graded alcohol series, and washed in distilled water. To enhance antigen retrieval, the sections were soaked in heat-induced sodium citrate buffer (pH 6.0) for 30 min, cooled to room temperature, and then incubated with 3% H_2_O_2_ in methanol for 15-20 min to block the endogenous peroxidase activity. After blocking, the sections were stained using a Histostain-SP Kit (AEC, Broad Spectrum) (859043, Life Technologies, Grand Island, NY) following the manufacturer’s instructions. Firstly, the sections were treated with a mouse anti-BVDV Npro polyclonal antibody (1:600) overnight at 4°C. After reacting with the primary antibody, the sections were stained with biotinylated anti-mouse IgG in the Kit for 15-20 min at 37°C. After washing, the color was developed with a DAB-containing substrate kit (ZLI-9018, ZSGB-BIO, Beijing, China) according to the manufacturer’s protocols. In addition, the tissue sections were stained with hematoxylin for 18 min at room temperature, washed, and stained with eosin for 11 min at room temperature. Then, the sections were examined under a light microscope.

### PBMC Preparation

PBMC were isolated from fresh-heparinized venous blood of mice by standard Ficoll/Hypaque density gradient centrifugation (Sigma, St. Louis, MO, USA). PBL and peripheral blood monocytes (PBM) were purified by adherent culture method and magnetic cell separation technique as described previously (21). Briefly, PBMC were incubated at 37°C for at least 2 h with 5% CO_2_. Non-adherent cells (lymphocytes) and adherent cells were individually washed twice in PBS. Adherent cells were incubated with mouse anti-bovine CD14 mAb (MCA2678F, Bio-Rad, CA, USA), followed by the addition of magnetic beads conjugated with mouse anti-IgG1 (130-047-101, Miltenyi Biotech, Auburn, CA). CD14^+^ monocytes were positively selected using the magnetic cell separation technique according to the manufacturer’s instructions (Miltenyi Biotech).

### Confocal Laser Scanning Microscopy Analysis of Virus-Infection

Splenic lymphocytes were separated by density gradient centrifugation (P8860, Solarbio, Beijing, China). PBL and splenic lymphocytes were collected by centrifugation at 1,000 g for 5 min at 4°C, washed once with PBS, and resuspended with 20 µL PBS. The suspension was dripped on poly-L-lysine-coated coverslip and dried at 37°C for 15 min. The cells were incubated with a mouse anti-BVDV Npro polyclonal antibody (1:100) overnight at 4°C, and then incubated with an CoraLite488-conjugated affinipure goat anti-mouse IgG (H+L) (SA00013-1, 1:200, Proteintech). A cell membrane fluorescent probe (FAST Dil) (abs47048140, Absin Bioscience, Shanghai, China) was used to stain the cell membranes. The nuclei were stained with 4′,6-diamidino-2-phenylindole (DAPI), and the coverslips were observed using a laser scanning confocal microscope (TCS SP2; Leica).

### Expression Analysis of PD-1 and PD-L1 mRNA During BVDV Infection

To study PD-1/PD-L1 mRNA expression after BVDV infection, total RNAs in PBL and PBM were extracted respectively using TRIzol reagent (Invitrogen). The mRNA expressions of PD-1 in PBL and PD-L1 in PBM at day 4, 7, and 10 of post-infection were measured using qRT-PCR with the CFX96 Touch Real-Time PCR Detection System (Bio-Rad, Hercules, CA, USA) using SYBR Premix Ex Taq II (RR820A, TaKaRa Biotechnology, Dalian, China). The primers used in qRT-PCR were 5’-GCAATC AGGGTGGCTTCT-3’ and 5’-TTGGCTCAAACCATTACAGA-3’ for mouse PD-1, 5’-AAGCCTCAGCACAGCAACTTCAG-3’ and 5’-TGTAGTCCGCACCACCGT AGC-3’ for mouse PD-L1, and 5’-TGCTGTCCCTGTATGCCTCT-3’ and 5’-TGT CACGCACGATTTCCC-3’ for internal control β-actin. The reaction cycling conditions were initial denaturation at 95°C for 30 s followed by 40 cycles of 95°C for 5 s, 60°C for 30 s, and 70°C for 30 s. A final melting curve analysis was performed from 65°C to 95°C at a rate of 0.1°C/s (continuous acquisition). Each sample was tested in triplicate, and the fold differences in gene expression were calculated using the 2^−ΔΔCt^ method with normalization to β-actin.

### Western Blot Analysis

The protein expressions of PD-1 in PBL and PD-L1 in PBM were measured by Western blot analysis at day 7 of post-infection. Total proteins were extracted respectively from PBL and PBM with 150 μL RIPA buffer (P0013B, Beytime, HangZhou, China) containing 15 mM PMSF (ST505, Beytime, China). Protein concentration was determined using the Enhanced BCA Protein Assay Kit (P0009, Beytime) according to the manufacturer’s instructions. Approximately 30 μg of total proteins were separated by sodium dodecyl sulfate-polyacrylamide gel electrophoresis (SDS-PAGE) and transferred onto polyvinylidene fluoride (PVDF) membranes (0.45 μm, Millipore, Germany). The membranes were blocked with 5% fat-free milk in TBST (Tris HCl, NaCl and Tween 20) for 1 h at room temperature, then incubated overnight at 4°C with primary antibodies against PD-1 (ab52587, 1:100, Abcam), PD-L1 (ab213524, 1:1000, Abcam), and β-actin (60008-1-lg, 1:15000, Proteintech), which was used as an internal control. After that, the membranes were rinsed with TBST five times for 10 min each and incubated with horseradish peroxidase (HRP)-conjugated Affinipure Goat Anti-Mouse IgG (H + L) (SA00001-1, 1:8000, Proteintech) or HRP-conjugated Affinipure Goat Anti-Rabbit IgG (H + L) (SA00001-2, 1:8000, Proteintech) for 1 h at room temperature. Then, the membranes were rinsed and treated with Chemiluminescent HRP Substrate (P90719, Millipore). The signals were detected with a chemiluminescence detector (Bio-Rad, USA), and the expression of each protein was measured with Image Lab software.

### PD-1 Blockade Assay

To assess the effects of PD-1 pathway on the proportions of T cell subsets, the apoptosis and proliferation of PBL from BVDV-infected mice, and the production of cytokines in serum, PD-1/PD-L1 interaction was blocked *in vivo* by anti-PD-1 antibody as described previously (22–24). The infected mice in each group were injected intraperitoneally with 200 μg of anti-PD-1 antibody (ICH1132, Ichorbio, Oxford, UK) or 200 μg of mouse IgG2a isotype control (ICH2244, Ichorbio, Oxford, UK) at day 1 of post-infection.

### Flow Cytometric Analysis Of CD3^+^, CD4^+^, and CD8^+^ T Cell Subsets

To study the effects of PD-1 blockade on the proportions of CD3^+^, CD4^+^, and CD8^+^ T cell subsets from BVDV-infected mice at day 7 of post-infection, PBMC were incubated with fluorescein isothiocyanate (FITC) - conjugated anti-CD3 monoclonal antibody (ab91493, Abcam, Cambridge, UK), allophycocyanin (APC) - conjugated anti-CD4 monoclonal antibody (ab252152, Abcam, Cambridge, UK) and phycoerythrin (PE) - conjugated anti-CD8 (ab272343, Abcam, Cambridge, UK) monoclonal antibody for 20 min at room temperature in the dark, washed twice with PBS, and resuspended with 200 µL PBS. The suspensions were analyzed immediately on a CytoFLEX flow cytometer (Beckman Coulter, USA).

### Flow Cytometric Analysis of Cell Apoptosis

To analyze the effects of PD-1 blockade on apoptosis of PBL from BVDV-infected mice at day 7 of post-infection, PBL were stained using Annexin-V-FITC apoptosis Kit with propidium iodide for 15 min at room temperature in the dark according to the manufacturer’s instructions (C1062M, Beyotime, Shanghae, China) and analyzed immediately on a CytoFLEX flow cytometer (Beckman Coulter, USA).

### Cell Proliferation Assay

To assess the effects of PD-1 blockade on the proliferation of PBL from BVDV-infected mice, PBMC in RPMI-1640 (Gibco, Carlsbad, CA, USA) were plated with 1 × 10^4^/well on flat-bottom 96-well microtiter plates and incubated at 37°C with 5% CO_2_ in the presence of 10 ng/mL phorbol 12-myristate acetate (PMA) and 500 ng/mL ionomycin (Sigma, St. Louis, MO, USA). The proliferation of PBL in each well was measured every 24 h till 168 h using Cell Counting Kit-8 (CK04, Dojindo Laboratories, Kumamoto, Japan) according to the manufacturer’s instructions.

### IL-2 and IFN-γ Measurement Using ELISA

To measure the effects of the PD-1 blockade on the production of IL-2 and IFN-γ at day 7 of post-infection, 400 μl of blood was collected from the mouse tail and centrifuged to obtain the serum. The production of IL-2 and IFN-γ in the serum was measured using a mouse IL-2 (SEA073Mu, USCN Life Science, Wuhan, China) and IFN-γ (SEA049Mu, USCN Life Science, Wuhan, China) ELISA kit according to the manufacturer’s protocol.

### Western Blot Analysis of PD-1 Downstream Signaling Molecules

PD-1 downstream signaling molecules in PBL from mice were measured by Western blot analysis at day 7 of post-infection. The primary antibodies were PI3K (ab227204, 1:1000, Abcam), p-PI3K (ab182651, 1:1000, Abcam), AKT (#9272, 1:1000, Cell Signaling Technology, USA), p-AKT (Ser473) (#9271, 1:1000, Cell Signaling Technology), ERK (#4695S, 1:1000, Cell Signaling Technology), p-ERK (Thr202/Tyr204) (#9101, 1:1000, Cell Signaling Technology), mTOR (ab2732, 1:1000, Abcam), p-mTOR (ab84400, 1:1000, Abcam), and β-actin (60008-1-lg, 1:15000, Proteintech), which was used as an internal control. The secondary antibodies were HRP-conjugated affinipure goat anti-mouse IgG (H+L) (SA00001-1, 1:8000, Proteintech) and HRP-conjugated affinipure goat anti-rabbit IgG (H+L) (SA00001-2, 1:8000, Proteintech).

### Statistical Analysis

All data were expressed as mean ± SD and analyzed using student’s unpaired t-test, one-way ANOVA, or two-way ANOVA using GraphPad Prism version 6.0 (GraphPad Software). A *p-value* less than 0.05 indicated a statistically significant difference. All samples were assayed in triplicate.

## Results

### BVDV-Infected Mice Showed No Clinical Signs of Illness

In this study, mice were infected with CP BVDV (strain NADL) and NCP BVDV (strain NY-1) by IP injection. As expected, none of the mice exhibited clinical signs of illness.

### BVDV Antigen Was Detected in the Main Organs of Infected Mice

**Figure 1:**
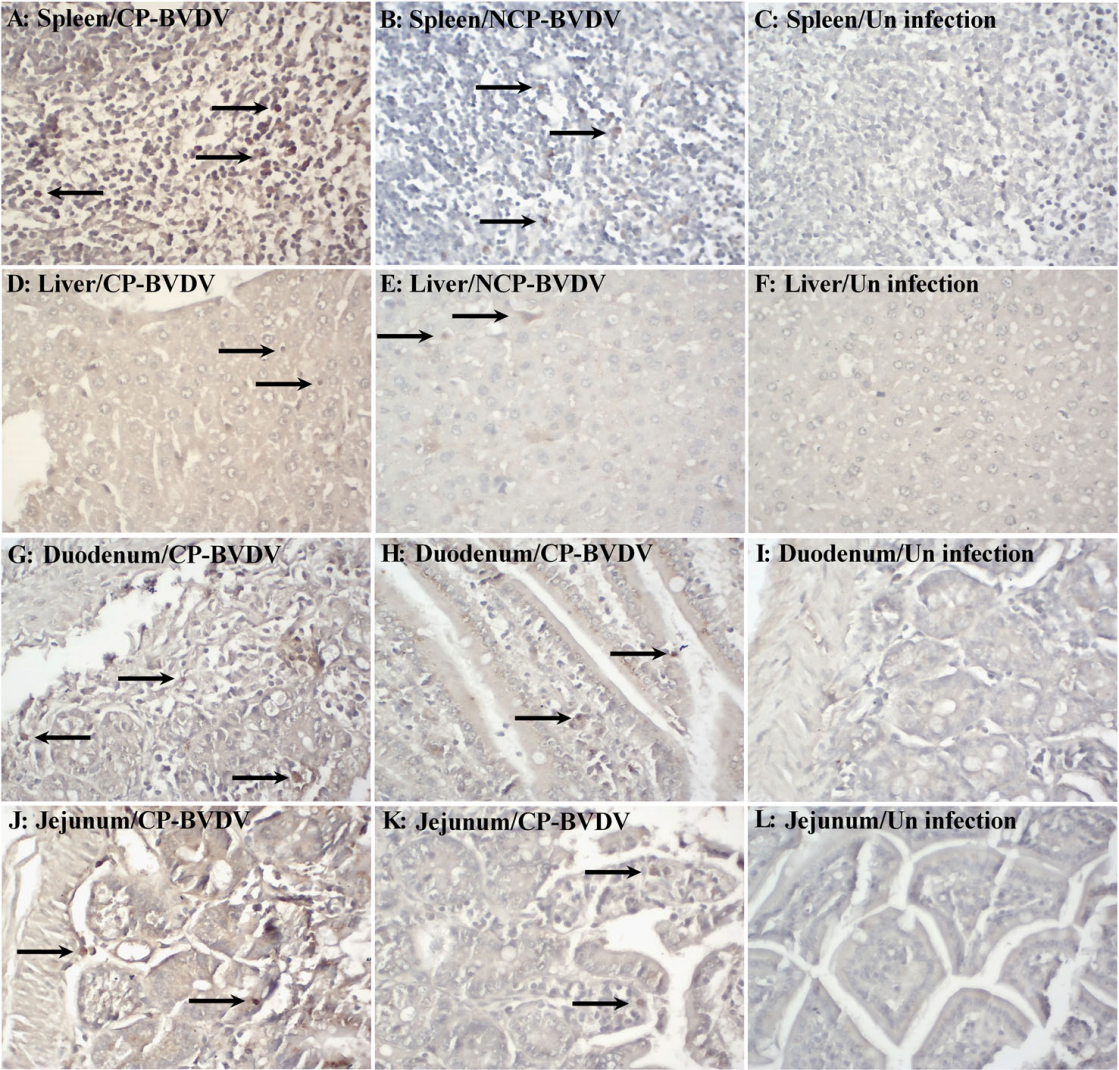
Immunohistochemistry for BVDV antigen at day 7 of post-infection. Viral antigen was detected in the spleen [**(A)** CP BVDV; **(B)** NCP BVDV], liver [**(D)** CP BVDV; **(E)** NCP BVDV], duodenum [**(G)** CP BVDV; **(H)** NCP BVDV], and jejunum [**(J)** CP BVDV; **(K)** NCP BVDV]. Images of the spleen **(C)**, liver **(F)**, duodenum **(I)**, and jejunum **(L)** of negative control mice are also shown (original magnification, 400×).

Based on IHC analysis of BVDV-infected mice, at day 7 of post-infection, the distribution of viral antigens in infected mice is summarized in **Table 1**. No BVDV antigen was detected in any tissue samples of mock-infected mice (**Figures 1C, F, I, L**). The detection rate of CP and NCP BVDV in the spleen (CP BVDV, 5/5, 100%, **Figure 1A**; NCP BVDV, 5/5, 100%, **Figure 1B**), liver (CP BVDV, 5/5, 100%, **Figure 1D**; NCP BVDV, 4/5, 80%, **Figure 1E**), duodenum (CP BVDV, 4/5, 80%, **Figure 1G**; NCP BVDV, 4/5, 80%, **Figure 1H**), and jejunum (CP BVDV, 5/5, 100%, **Figure 1J**; NCP BVDV, 4/5, 80%, **Figure 1K**) samples reached more than 80%.

**Table 1:** Distribution of BVDV-specific antigen at day 7 of post-infection in the five mice infected with each BVDV strain.

	Heart	Lung	Liver	Spleen	Duodenum	Jejunum	Ileum	Colon
CP BVDV	3/5	3/5	5/5	5/5	4/5	5/5	3/5	2/5
NCP BVDV	3/5	3/5	4/5	5/5	4/5	4/5	3/5	3/5
Control	0/5	0/5	0/5	0/5	0/5	0/5	0/5	0/5

### BVDV Infection Caused Histopathological Lesions in Mice

**Figure 2:**
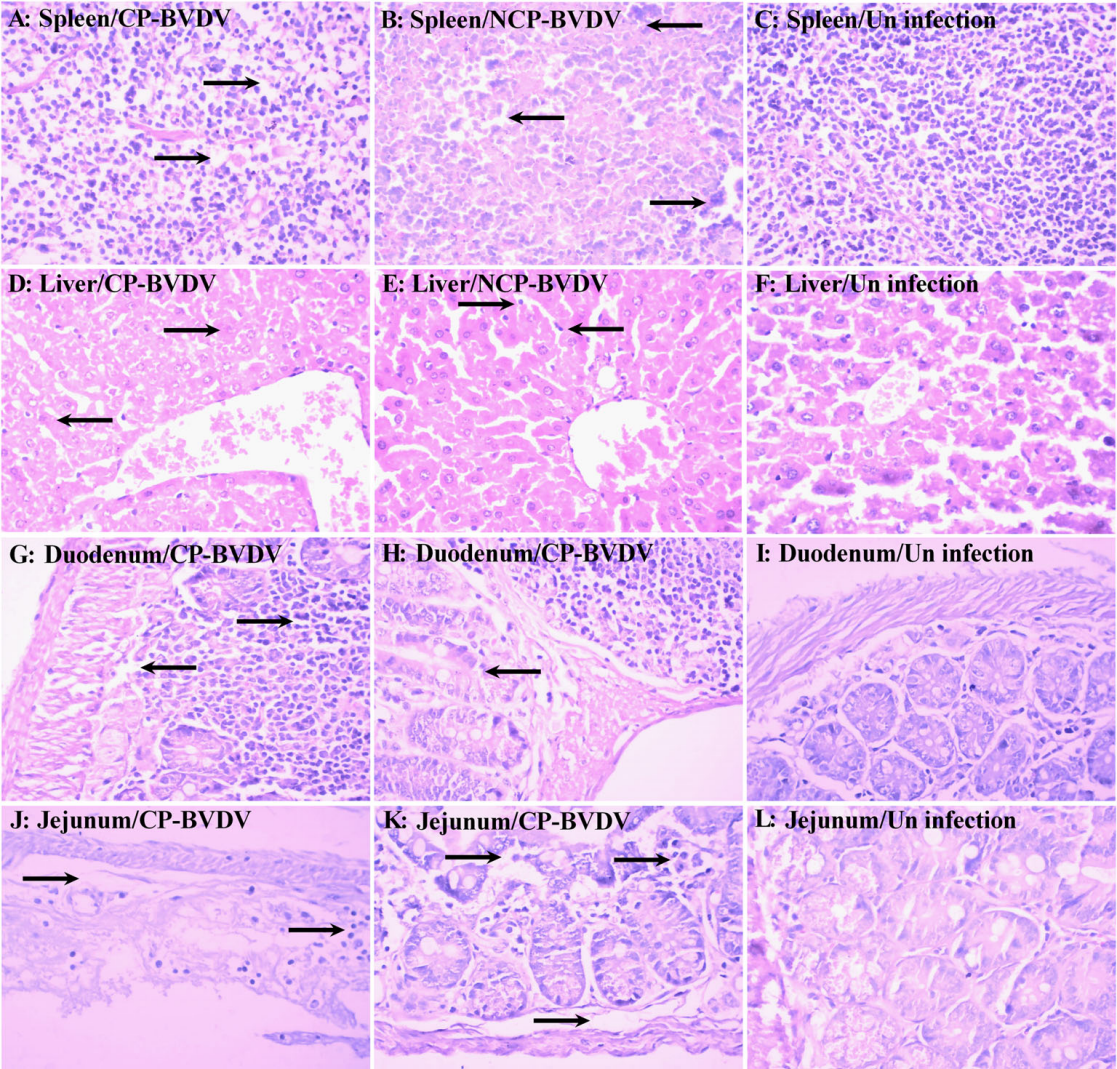
Histopathology of the spleen, liver, duodenum, and jejunum at day 7 of post-infection. Histological lesions were detected in the spleen [**(A)** CP BVDV; **(B)** NCP BVDV], liver [**(D)** CP BVDV; **(E)** NCP BVDV], duodenum [**(G)** CP BVDV; **(H)** NCP BVDV], and jejunum [**(J)** CP BVDV; **(K)** NCP BVDV]. Images of the spleen [**(C)**, liver **(F)**, duodenum **(I)**, and jejunum **(L)** of a negative control mouse are also shown (Hematoxylin and eosin, original magnification, 400×).

Histopathological examinations were performed on the spleen, liver, duodenum, and jejunum of the infected mice at day 7 of post-infection. Lymphocyte degeneration and necrosis and interstitial looseness and edema were observed in the spleens of mice infected with CP (**Figure 2A**) and NCP (**Figure 2B**) BVDV. Hepatocyte swelling, vacuolar degeneration, and necrosis were observed in the liver of mice infected with CP BVDV (**Figure 2D**), while lymphocyte infiltration of hepatic sinusoids was observed in the liver of mice infected with NCP BVDV (**Figure 2E**). The duodenums of CP BVDV-infected mice showed the degeneration, necrosis, and shedding of mucosal epithelial cells, interstitial looseness and edema, and inflammatory cell infiltration (**Figure 2G**), while the duodenums of NCP BVDV-infected mice showed the degeneration, necrosis, and shedding of intestinal glandular epithelium, interstitial looseness and edema, and inflammatory cell infiltration (**Figure 2H**). Shedding of mucosal epithelial cells, looseness, and edema of lamina propria, and inflammatory cell infiltration were found in the jejunum of mice infected with CP BVDV (**Figure 2J**), while necrosis of intestinal epithelial cells and inflammatory cell infiltration were observed in the jejunum of mice infected with NCP BVDV (**Figure 2K**). No significant histological lesions were seen in mock-infected mice (**Figures 2C, F, I, L**).

### PBL and Splenic Lymphocytes Were Infected by BVDV

**Figure 3:**
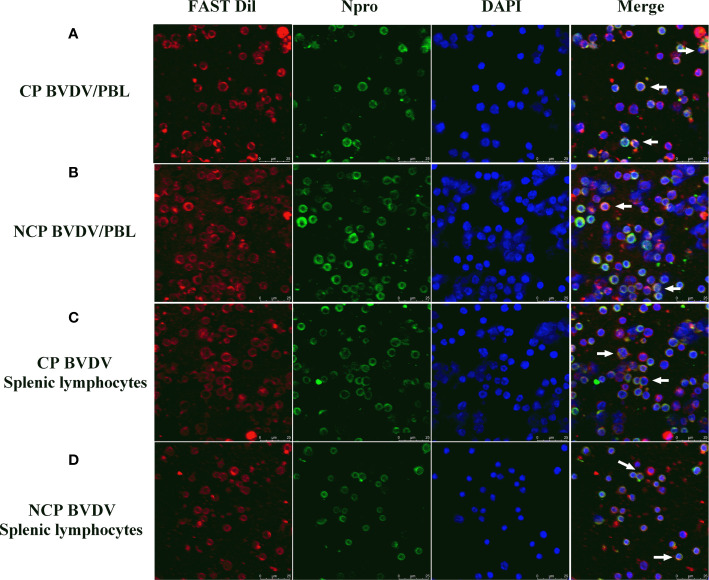
CLSM analysis of virus-infected PBL and splenic lymphocytes at day 7 of post-infection. **(A)** CLSM analysis of CP BVDV-infected PBL. **(B)** CLSM analysis of NCP BVDV-infected PBL. **(C)** CLSM analysis of CP BVDV-infected splenic lymphocytes. **(D)** CLSM analysis of NCP BVDV-infected splenic lymphocytes. The green color denotes BVDV Npro, the red color denotes the cell membranes and the blue color denotes DAPI. Scale bar: 25 μm.

To determine if PBL and splenic lymphocytes were infected by the virus, PBL and splenic lymphocytes from BVDV-infected mice were analyzed by CLSM at day 7 of post-infection. Observation using CLSM intuitively showed that PBL (CP BVDV, **Figure 3A**; NCP BVDV, **Figure 3B**) and splenic lymphocytes (CP BVDV, **Figure 3C**; NCP BVDV, **Figure 3D**) were infected by virus at day 7 of post-infection.

### Expression of PD-1 and PD-L1 mRNA Was Increased in BVDV-Infected Mice

**Figure 4:**
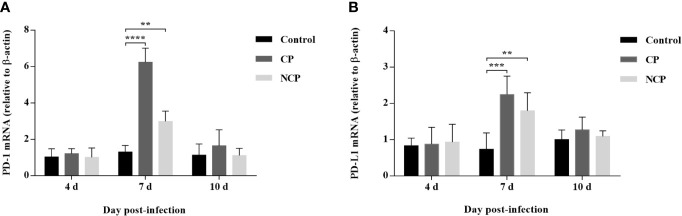
Quantitative analysis of mRNA expression of PD-1 and PD-L1 during BVDV infection. **(A)** The expression and quantification of PD-1 mRNA in PBL. **(B)** The expression and quantification of PD-L1 mRNA in PBM. ****p < 0.0001, ***p < 0.001, **p < 0.01. Mock-infected mice were used as the control group. Data are presented as mean ± SD (n = 5 per group) and analyzed using two-way ANOVA.

Our data showed that the level of PD-1 mRNA in PBL from BVDV-infected mice was significantly upregulated compared with mock-infected mice at day 7 of post-infection (CP BVDV, *p* < 0.0001, **Figure 4A**; NCP BVDV, *p* < 0.01, **Figure 4A**). Meanwhile, the PD-L1 mRNA expression was significantly increased in PBM from the BVDV-infected mice than in PBM from the mock-infected mice at day 7 of post-infection (CP BVDV, *p* < 0.001, **Figure 4B**; NCP BVDV, *p* < 0.01, **Figure 4B**).

### Expression of PD-1 and PD-L1 Protein Was Upregulated in BVDV-Infected Mice

**Figure 5:**
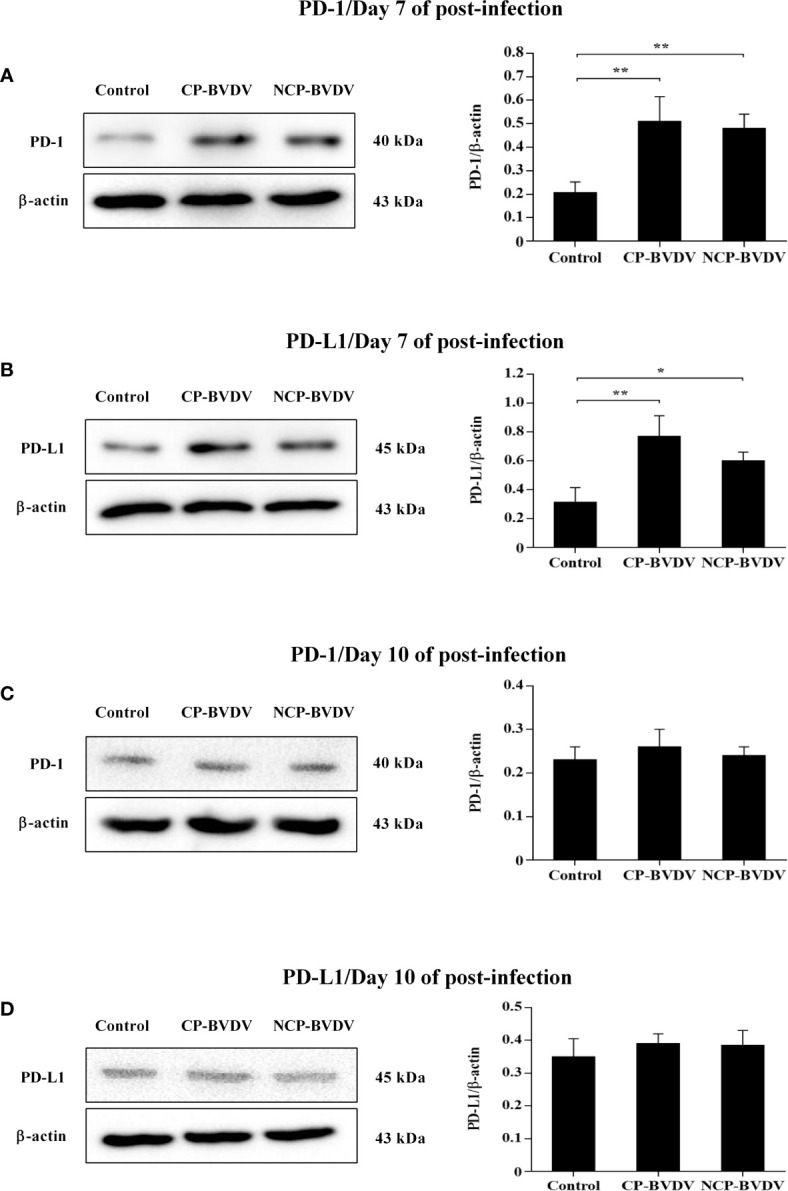
Western blot analysis of PD-1 and PD-L1 protein expression at days 7 and 10 of post-infection. **(A)** The expression of PD-1 protein in PBL at day 7 of post-infection. **(B)** The expression of PD-L1 protein in PBM at day 7 of post-infection. **(C)** The expression of PD-1 protein in PBL at day 10 of post-infection. **(D)** The expression of PD-L1 protein in PBM at day 10 of post-infection. **p < 0.01, *p < 0.05. Mock-infected mice were used as the control group. Data are presented as mean ± SD (n = 5 per group) and analyzed using one-way ANOVA.

Western blot results showed that the PD-1 expression was significantly upregulated in PBL from CP (*p* < 0.01, **Figure 5A**) and NCP (*p* < 0.01, **Figure 5A**) BVDV-infected mice at day 7 of post-infection compared with mock-infected mice. Likewise, the PD-L1 expression was significantly upregulated in PBM from CP (*p* < 0.01, **Figure 5B**) and NCP (*p* < 0.05, **Figure 5B**) BVDV-infected mice at day 7 of post-infection compared with mock-infected mice. In addition, CP and NCP BVDV infection did not significantly affect PD-1 and PD-L1 expression on day 10, as shown in **Figures 5C, D**. Original data is shown in **Supplementary Material**.

### PD-1 Blockade Restored Weight Gain in BVDV-Infected Mice

**Figure 6:**
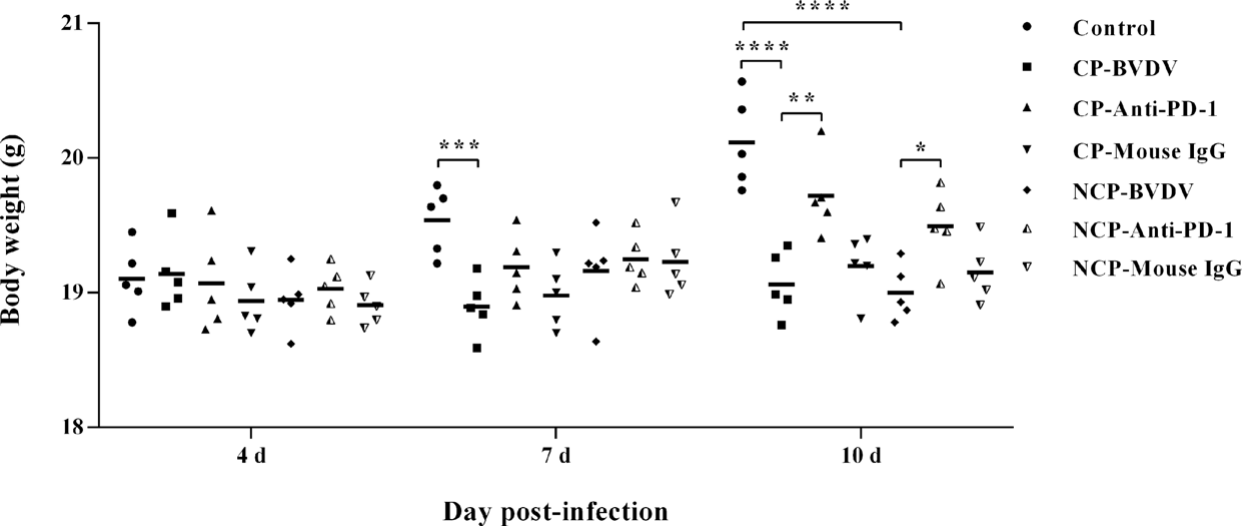
Changes in body weight of mice in BVDV-infected groups and the mock-infected groups. ****p < 0.0001, ***p < 0.001, **p < 0.01, *p < 0.05. Mock-infected mice were used as the control group. Data are presented as mean ± SD (n = 5 per group) and analyzed using two-way ANOVA.

We observed body weight changes during the experiment. As shown in **Figure 6**, with the extension of experiment time, the body weight of mice in the mock-infected groups increased gradually, while the body weight of mice infected with CP and NCP BVDV remained basically unchanged. In addition, the body weight of the BVDV-infected mice decreased significantly at day 7 (CP BVDV, *p* < 0.001) and 10 (CP BVDV, *p* < 0.0001; NCP BVDV, *p* < 0.0001) of post-infection compared with the mock-infected mice. Notably, PD-1 blockade significantly increased the body weight of mice in the BVDV-infected groups at day 10 (CP BVDV, *p* < 0.01; NCP BVDV, *p* < 0.05) of post-infection. In addition, there was no significant change in body weight in the IgG antibody treated mice compared with BVDV-infected mice.

### PD-1 Blockade Increased the Number of White Blood Cell and Lymphocyte in the Blood of BVDV-Infected Mice

**Figure 7:**
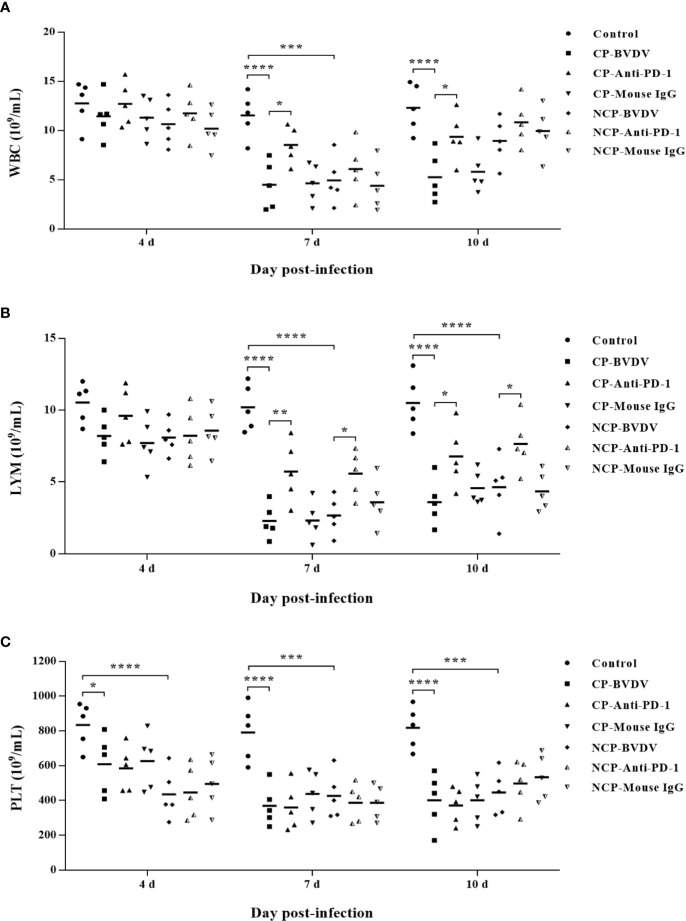
Hematological changes in BVDV-infected mice and mock-infected mice. **(A)** white blood cells (WBC), **(B)** lymphocytes (LYM), **(C)** platelets (PLT). ****p < 0.0001, ***p < 0.001, *p < 0.05. Mock-infected mice were used as the control group. Data are presented as mean ± SD (n = 5 per group) and analyzed using two-way ANOVA.

Hematological assessment showed that leukopenia was observed in BVDV-infected mice at day 7 (CP BVDV, *p* < 0.0001, **Figure 7A**; NCP BVDV, *p* < 0.001, **Figure 7A**) and 10 (CP BVDV, *p* < 0.0001, **Figure 7A**) of post-infection. Moreover, lymphopenia was also found in BVDV-infected mice at day 7 (CP BVDV, *p* < 0.0001, **Figure 7B**; NCP BVDV, *p* < 0.0001, **Figure 7B**) and 10 (CP BVDV, *p* < 0.0001, **Figure 7B**; NCP BVDV, *p* < 0.0001, **Figure 7B**) of post-infection. Platelet counts showed a significant reduction in all BVDV-infected mice at day 4 (CP BVDV, *p* < 0.05, **Figure 7C**; NCP BVDV, *p* < 0.0001, **Figure 7C**), 7 (CP BVDV, *p* < 0.0001, **Figure 7C**; NCP BVDV, *p* < 0.001, **Figure 7C**) and 10 (CP BVDV, *p* < 0.0001, **Figure 7C**; NCP BVDV, *p* < 0.001, **Figure 7C**) of post-infection. Remarkably, PD-1 blockade significantly increased the number of white blood cell in BVDV-infected mice at day 7 (CP BVDV, *p* < 0.05, **Figure 7A**) and 10 (CP BVDV, *p* < 0.05, **Figure 7A**) of post-infection. Meanwhile PD-1 blockade significantly increased the lymphocyte number in BVDV-infected mice at day 7 (CP BVDV, *p* < 0.01, **Figure 7B**; NCP BVDV, *p* < 0.05, **Figure 7B**) and 10 (CP BVDV, *p* < 0.05, **Figure 7B**; NCP BVDV, *p* < 0.05, **Figure 7B**) of post-infection.

### PD-1 Blockade Increased Proportions of CD3^+^, CD4^+^, and CD8^+^ T Cell Subsets

**Figure 8:**
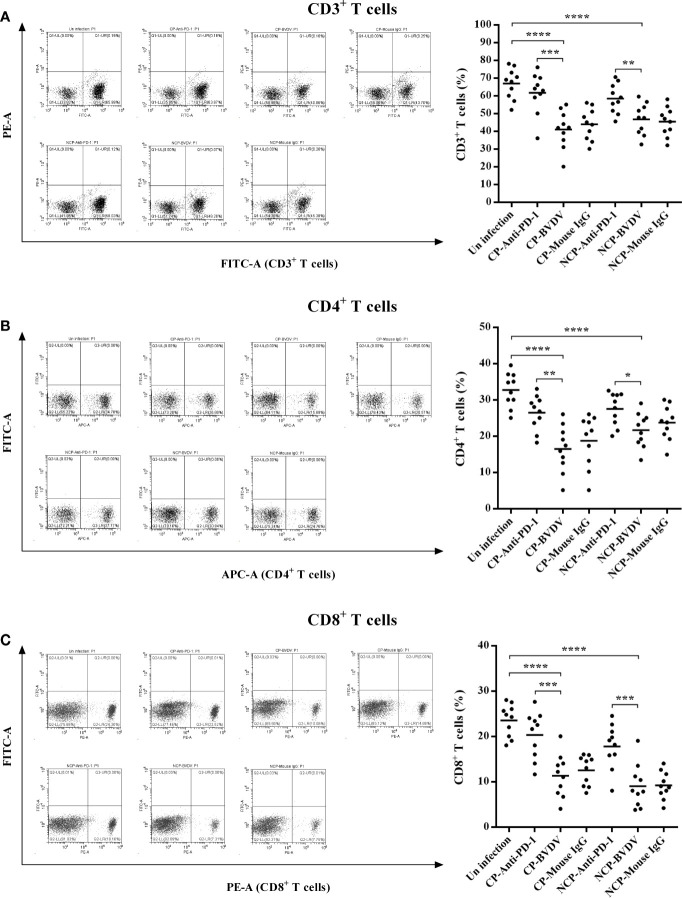
Effect of PD-1 blockade on the proportions of CD3^+^, CD4^+^, and CD8^+^ T cell subsets at day 7 of post-infection. **(A)** CD3^+^ T cells, **(B)** CD4^+^ T cells, **(C)** CD8^+^ T cells. ****p < 0.0001, ***p < 0.001, **p < 0.01, *p < 0.05. The animals were assigned into 7 experimental groups, including the mock-infected group, CP BVDV-infected group, CP BVDV+anti-PD-1 group, CP BVDV+mouse IgG group, NCP BVDV-infected group, NCP BVDV+anti-PD-1 group, and NCP BVDV+mouse IgG group. CP and NCP BVDV-infected mice were used as the controls. Data are presented as mean ± SD (n = 10 per group) and analyzed using one-way ANOVA.

Flow cytometric analysis showed that BVDV-infected mice had reduced percentage of CD3^+^ (CP BVDV, *p* < 0.0001, **Figure 8A**; NCP BVDV, *p* < 0.0001, **Figure 8A**), CD4^+^ (CP BVDV, *p* < 0.0001, **Figure 8B**; NCP BVDV, *p* < 0.0001, **Figure 8B**), and CD8^+^ (CP BVDV, *p* < 0.0001, **Figure 8C**; NCP BVDV, *p* < 0.0001, **Figure 8C**) T cells at day 7 of post-infection compared to the mock-infected mice. Remarkably, PD-1 blockade significantly increased the proportion of CD3^+^ (CP BVDV, *p* < 0.001, **Figure 8A**; NCP BVDV, *p* < 0.01, **Figure 8A**), CD4^+^ (CP BVDV, *p* < 0.01, **Figure 8B**; NCP BVDV, *p* < 0.05, **Figure 8B**), and CD8^+^ (CP BVDV, *p* < 0.001, **Figure 8C**; NCP BVDV, *p* < 0.001, **Figure 8C**) T cells in BVDV-infected mice.

### PD-1 Blockade Decreased The Apoptosis of PBL in BVDV-Infected Mice

**Figure 9:**
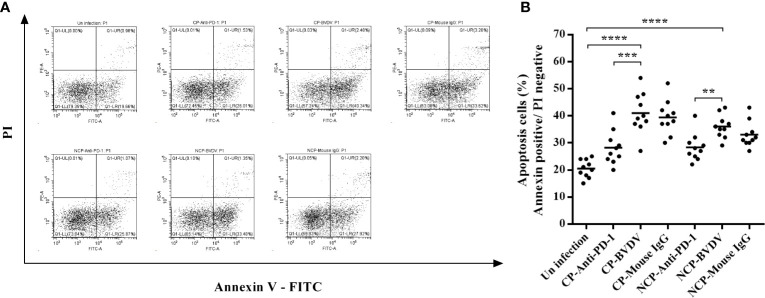
Effect of PD-1 blockade on apoptosis of PBL from the BVDV-infected mice at day 7 of post-infection. **(A)** Flow cytometry analysis of apoptosis of PBL. **(B)** Statistical analysis of apoptosis of PBL. ****p < 0.0001, ***p < 0.001, **p < 0.01. The animals were assigned into 7 experimental groups, including the mock-infected group, CP BVDV-infected group, CP BVDV+anti-PD-1 group, CP BVDV+mouse IgG group, NCP BVDV-infected group, NCP BVDV+anti-PD-1 group, and NCP BVDV+mouse IgG group. CP and NCP BVDV-infected mice were used as the controls. Data are presented as mean ± SD (n = 10 per group) and analyzed using one-way ANOVA.

Flow cytometric analysis showed that both CP (*p* < 0.0001) and NCP (*p* < 0.0001) BVDV infection led to a significant increase in the apoptosis of PBL in mice at day 7 of post-infection (**Figure 9**). Notably, PD-1 blockade significantly decreased the apoptosis of PBL in BVDV-infected mice (CP BVDV, *p* < 0.001, **Figure 9B**; NCP BVDV, *p* < 0.01, **Figure 9B**).

### PD-1 Blockade Increased PBL Proliferation in BVDV-Infected Mice

**Figure 10:**
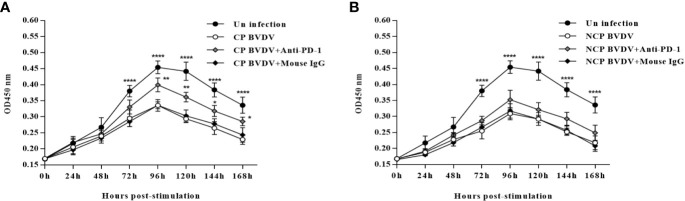
Effect of PD-1 blockade on the proliferation of PBL from BVDV-infected mice from 0 h to 168 h post-infection. **(A)** Proliferation of PBL from CP BVDV-infected mice. **(B)** Proliferation of PBL from NCP BVDV-infected mice. ****p < 0.0001, **p < 0.01, *p < 0.05. The animals were assigned into 7 experimental groups, including the mock-infected group, CP BVDV-infected group, CP BVDV+anti-PD-1 group, CP BVDV+mouse IgG group, NCP BVDV-infected group, NCP BVDV+anti-PD-1 group, and NCP BVDV+mouse IgG group. CP and NCP BVDV-infected mice were used as the controls. OD450 nm is proportional to the number of living cells and represents the level of PBL proliferation. Data are presented as mean ± SD (n = 10 per group) and analyzed using two-way ANOVA.

In this study, we confirmed that both CP BVDV (**Figure 10A**) and NCP BVDV (**Figure 10B**) inhibited PBL proliferation, and this inhibition was restored by the PD-1 blockade. In CP BVDV-infected mice, PD-1 blockade significantly increased PBL proliferation from 72 to 168 h of post-infection (**Figure 10A**). However, in NCP BVDV-infected mice, PD-1 blockade did not significantly restore PBL proliferation (**Figure 10B**).

### PD-1 Blocking Increased IFN-γ Production in the Serum From CP BVDV-Infected Mice

**Figure 11:**
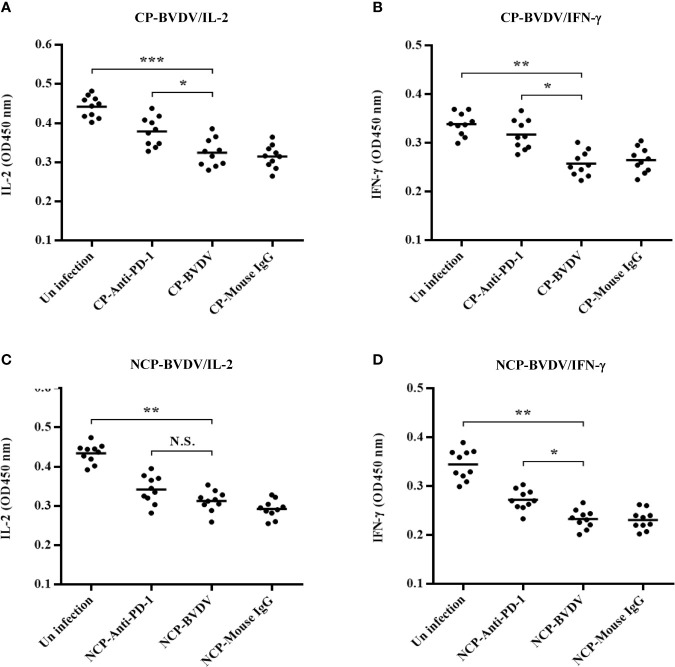
Effect of PD-1 blockade on IL-2 and IFN-γ in the serum of BVDV-infected mice at day 7 of post-infection. **(A)** IL-2 production in the serum of CP BVDV-infected mice. **(B)** IFN-γ production in the serum of CP BVDV-infected mice. **(C)** IL-2 production in the serum of NCP BVDV-infected mice. **(D)** IFN-γ production in the serum of NCP BVDV-infected mice. ***p < 0.001, **p < 0.01, *p < 0.05, NS, not significant. The animals were assigned into 7 experimental groups, including the mock-infected group, CP BVDV-infected group, CP BVDV+anti-PD-1 group, CP BVDV+mouse IgG group, NCP BVDV-infected group, NCP BVDV+anti-PD-1 group, and NCP BVDV+mouse IgG group. CP and NCP BVDV-infected mice were used as the controls. Data are presented as mean ± SD (n = 10 per group) and analyzed using one-way ANOVA.

In this study, IL-2 and IFN-γ were measured in the serum using ELISA. PD-1 blocking significantly increased IL-2 (*p* < 0.05, **Figure 11A**) and IFN-γ (*p* < 0.05, **Figure 11B**) production in the serum from CP BVDV-infected mice at day 7 of post-infection. Additionally, PD-1 blockade also significantly increased serum IFN-γ (*p* < 0.05, **Figure 11D**) production in NCP BVDV-infected mice but did not notably alter serum IL-2 production (**Figure 11C**).

### PD-1 Blockade Inhibited Viral Replication in Mice

**Figure 12:**
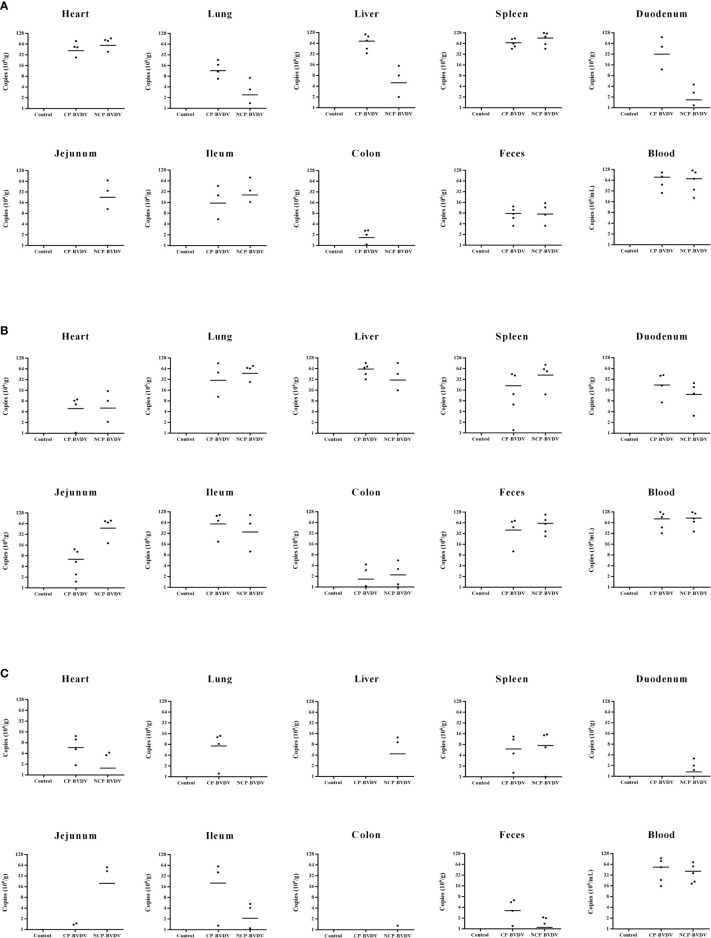
Copy numbers of virus gene in the BVDV-infected mice. **(A)** day 4 of post-infection, **(B)** day 7 of post-infection, **(C)** day 10 of post-infection. Mock-infected mice were used as the control group. Data are presented as mean ± SD (n = 5 per group). The unit of data for the Y axis in mouse tissues and feces is copies/g, and in blood is copies/mL.

**Figure 13:**
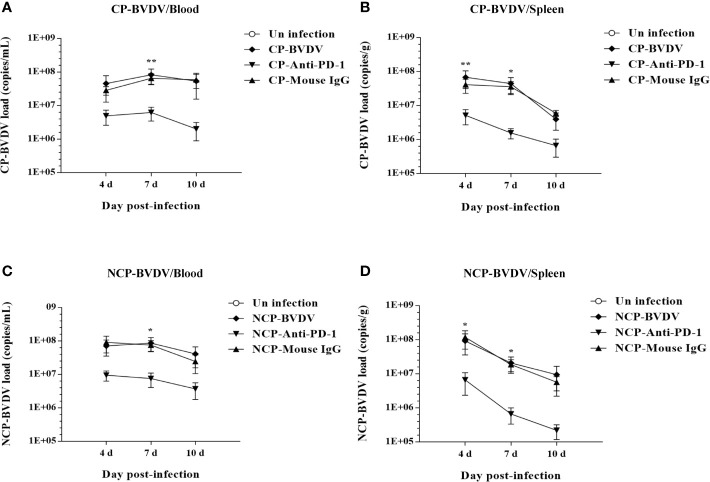
Effect of PD-1 blockade on virus replication in the blood and spleen of BVDV-infected mice at day 7 of post-infection. **(A)** the replication of the CP BVDV in blood. **(B)** the replication of the CP BVDV in spleen. **(C)** the replication of the NCP BVDV in blood. **(D)** the replication of the NCP BVDV in spleen. **p < 0.01, *p < 0.05. The animals were assigned into 7 experimental groups, including the mock-infected group, CP BVDV-infected group, CP BVDV+anti-PD-1 group, CP BVDV+mouse IgG group, NCP BVDV-infected group, NCP BVDV+anti-PD-1 group, and NCP BVDV+mouse IgG group. CP and NCP BVDV-infected mice were used as the controls. Data are presented as mean ± SD (n = 5 per group) and analyzed using two-way ANOVA.

To determine the viral replication in mouse tissues, feces, and blood, we detected the copy number of viral genes by qRT-PCR. At day 4 of post-infection, the copy number could be detected in all samples except in jejunal samples of CP BVDV-infected mice and in all samples except colon samples of NCP BVDV-infected mice (**Figure 12A**). On day 7 of post-infection, the copy number was detected in all samples of CP BVDV-infected and NCP BVDV-infected mice (**Figure 12B**). On day 10 of post-infection, the copy number could be detected in all samples except liver, duodenum, and colon samples in CP BVDV-infected mice and in all samples except lung samples in NCP BVDV-infected mice (**Figure 12C**). Both CP and NCP BVDV-infected mice had high copy numbers in spleen and blood samples throughout the experimental period (**Figure 12**). With the prolongation of infection time, the copy number in spleen samples of both CP and NCP BVDV-infected mice decreased gradually, while the copy number in lung, ileum and feces samples increased first before decrease (**Figure 12**). Remarkably, after treatment with an anti-PD-1 antibody, the replication of the CP BVDV (*p* < 0.01, **Figure 13A**) and NCP BVDV (*p* < 0.05, **Figure 13C**) were significantly inhibited at day 7 of post-infection in blood. In addition, in spleen, PD-1 blockade significantly inhibited the replication of the CP BVDV at day 4 (*p* < 0.01, **Figure 13B**) and 7 (*p* < 0.05, **Figure 13B**) of post-infection and the replication of the NCP BVDV at day 4 (*p* < 0.05, **Figure 13D**) and 7 (*p* < 0.05, **Figure 13D**) of post-infection.

### PD- 1 Blockade Upregulated the Expression Levels of p-mTOR and p-ERK in the CP BVDV Infection

**Figure 14:**
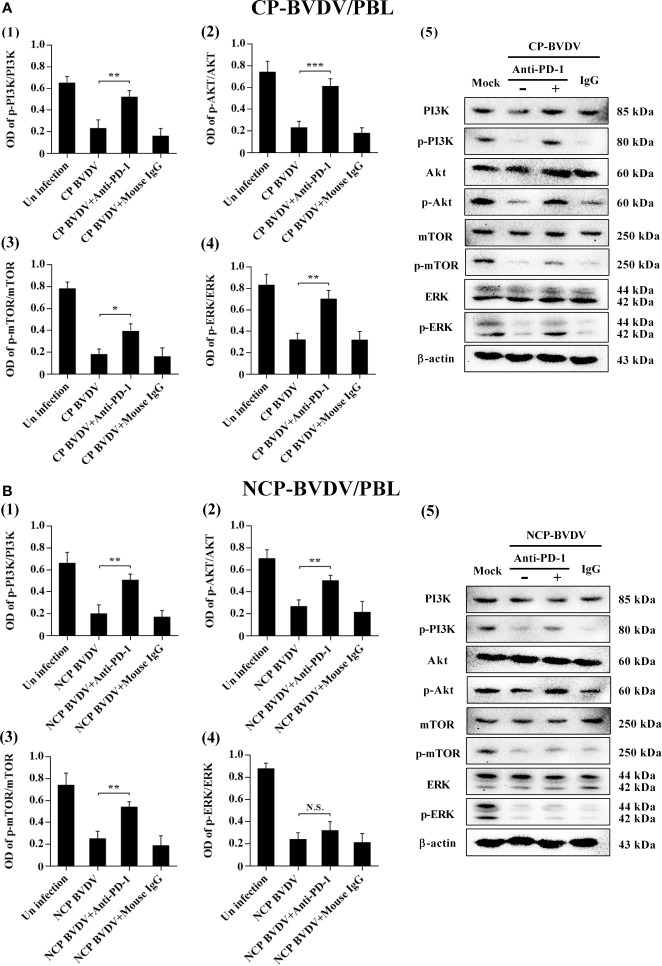
Effect of PD-1 blockade on the PD-1 downstream signaling molecules in PBL from BVDV-infected mice. **(A)** shown are the results of densitometric analyses of the levels of p-PI3K (1), p-Akt (2), p-mTOR (3), p-ERK (4) in bar graph format as well as the representative results (5) of Western blot analysis of PI3K, p-PI3K, Akt, p-Akt, mTOR, p-mTOR, ERK, p-ERK, and β-actin in CP BVDV-infected mice. **(B)** shown are the results of densitometric analyses of the levels of p-PI3K (1), p-Akt (2), p-mTOR (3), p-ERK (4) in bar graph format as well as the representative results (5) of Western blot analysis of PI3K, p-PI3K, Akt, p-Akt, mTOR, p-mTOR, ERK, p-ERK, and β-actin in NCP BVDV-infected mice. ***p < 0.001, **p < 0.01, *p < 0.05, NS, not significant. The animals were assigned into 7 experimental groups, including the mock-infected group, CP BVDV-infected group, CP BVDV+anti-PD-1 group, CP BVDV+mouse IgG group, NCP BVDV-infected group, NCP BVDV+anti-PD-1 group, and NCP BVDV+mouse IgG group. CP and NCP BVDV-infected mice were used as the controls. Data are presented as mean ± SD (n = 5 per group) and analyzed using one-way ANOVA.

To determine the effects of PD-1 blockade on the expression and phosphorylation of downstream signaling molecules, western blot analysis was performed. In the CP BVDV-infected mice, the expression levels of p-PI3K, p-Akt, p-mTOR and p-ERK in PBL were significantly upregulated by PD-1 blockade (**Figures 14A**). Furthermore, in the NCP BVDV-infected mice, we observed a significant increase in p-PI3K, p-Akt and p-mTOR (**Figures 14B**). Remarkably, PD-1 blockade had no significant effect on p-ERK expression in PBL from the NCP BVDV-infected mice (**Figures 14B**).

## Discussion

To further investigate the immunopathological mechanisms of BVDV *in vivo*, suitable small animal models are essential and may serve as useful tools. Previous research (19) showed that BVDV infection in mice was successfully induced by IP and demonstrated the potential of mice as a model for BVDV infection, although none of the IP-injected mice in that study exhibited any signs of illness. In this study, mice were infected with CP BVDV (strain NADL) and NCP BVDV (strain NY-1) by IP injection. Consistent with the results of the previous studies, we found none of the mice exhibited clinical signs of illness other than a significant difference in body weight. In addition, acute BVDV infection in cattle is characterized by peripheral blood leukopenia, lymphopenia, and thrombocytopenia (7, 25). A previous study (26) demonstrated that leukopenia, lymphocytopenia, and thrombocytopenia were observed in CP BVDV-infected mice. Similarly, in this study, we also observed leukopenia, lymphocytopenia, and thrombocytopenia in both CP and NCP BVDV-infected mice. These results suggest that leukopenia, lymphocytopenia, and thrombocytopenia may be one of the important indicators for the reliable establishment of animal models of BVDV infection, which provides a basis for exploring the immunopathological mechanisms of lymphocytopenia and immunosuppression caused by BVDV and other related viruses *in vivo*.

In the distribution of viral antigens, the previous study (20) showed that viral antigens were detected in the spleen of all infected mice from days 4 through 14 of post-infection and suggested that the spleen is the most reliable tissue for BVDV antigen detection in a murine model. In the present study, we found that viral antigens were consistently detected in both the spleen and blood. The presence of viremia provided evidence that the mice were infected with BVDV. Notably, a limitation of our study is that the co-staining of cells and virus antigen were not detected during the experiment and needs to better evaluate by a more visual method, such as immunofluorescence. In this study, CP-BVDV was not detectable in Jejunum but in both Duodenum and Ileum. NCP-BVDV was not detectable in colon but in feces. In fact, we observed the difference in virus distribution in different tissues at day 4 of post-infection, which is consistent with previous reports (20, 27). The difference may be related to the tissue tropism or cell tropism of BVDV and the time course of virus infection (27, 28). In addition, we found that BVDV become undetetable in many tissues but still high in the blood at day 10 of post-infection. BVDV are highly lymphotrophic (28). Peripheral blood lymphocytes and monocytes are susceptible to BVDV infection to produce progeny viruses (18, 29). In acute BVDV infection, viruses replicate in peripheral blood lymphocytes, associated lymphoid tissues, and mucosal epithelium, leading to viraemia and subsequent viral replication and leukopenia in different organ systems throughout the body (7, 30). BVDV may invade the circulation system and induce the viremia. Then, specific neutralizing antibodies appeared from 10-14 days after infection. It could be that these antibodies neutralized the virus in the tissues. Moreover, the mice infected by IP injection showed histopathological lesions in the spleen, liver, duodenum, and jejunum of the infected mice on day 7 of post-infection in this study. Overall, our results provided evidence of CP (strain NADL) and NCP (strain NY-1) BVDV infection in mice after IP injection.

PD-1 plays a crucial role in immunomodulation (31), and up-regulated PD-1 expression is generally associated with lymphopenia and apoptosis in many viral infections, such as HIV (32) and HCV (33). In a previous study, we reported that PD-1 plays a vital role in peripheral blood lymphopenia and apoptosis caused by acute BVDV infection *in vitro* (17). Meanwhile, PD-1 blockade inhibits PBL apoptosis and restores proliferation and anti-viral immune functions of PBL. However, the *in vivo* situation remains to be further studied and confirmed. In the present study, we further demonstrated the immunomodulatory role of PD-1 in peripheral blood lymphocytopenia in a mouse model of BVDV infection. About the expression of PD-1, previous studies showed that PD-1 and PD-L1 expression levels were correlated with viral load in the progress of several diseases, such as hepatitis C and AIDS (11, 34). Moreover, PD-1 and PD-1 expression was positively correlated with viral load in CSFV infection (35). CSFV also belong to the genus Pestivirus of the Flaviviridae family along with BVDV. PD-1 expression was significantly up-regulated during CSFV infection and at day 7 of post-infection, consistent with HCV and HIV infection (36, 37). Meanwhile, CSFV load in blood was up to the highest at 7 days of post-infection. In this study, we observed similar results in BVDV. Notably, previous studies showed that both HCV-core protein and HIV-1 accessory protein Nef could induce PD-1 expression (38, 39). Consequently, BVDV infection certainly contributes to the upregulation of PD-1 and PD-L1 levels during acute BVDV infection, although it is impossible to conclude that PD-1 and PD-L1 levels were modulated by undefined proteins of BVDV from current data. In addition, previous studies (40, 41) have confirmed a decrease in the proportion of CD4^+^ and CD8^+^ T cells in BVDV-infected cattle. In this study, we also found a decrease in the percentage of CD3^+^, CD4^+^, and CD8^+^ T cells in the peripheral blood of CP and NCP BVDV-infected mice and PD-1 blockade could restore the percentage of CD3^+^, CD4^+^, and CD8^+^ T cells and inhibit PBL apoptosis. Our study provides a basis for future studies on the immunomodulatory effects of the PD-1 pathway on major PBL subsets *in vivo*.

In this study, we found that PD-1 blockade restores PBL proliferation and IL-2 production in CP BVDV-infected mice, but not CP BVDV-infected mice. A previous paper, published in Archives of Virology, showed that enhanced ERK phosphorylation was detected following infection with CP BVDV, but not with NCP BVDV (42). Notably, ERK is an important signaling molecule downstream of PD-1 pathway and can regulate cell proliferation and IL-2 production. PD-1 blockade restored lymphocyte proliferation by reactivating the ERK pathway (43). Particularly, our previous study confirmed *in vitro* that PD-1 blockade significantly increased lymphocyte proliferation and p-ERK level after CP BVDV infection but did not after NCP BVDV infection. The difference in the effect of PD-1 blockade on lymphocyte proliferation and IL-2 production in CP and NCP BVDV infection may be related to the different effects of the two viruses on the ERK pathway after infection.

IFN-γ is a critical cytokine that plays an essential role in both innate and adaptive immunity and antiviral processes (44). It has been determined that PD-1 blockade by specific antibodies would upregulate IFN-γ production and restore antiviral immunity (13, 45). In this study, IFN-γ production was also increased by PD-1 blockade, consistent with the results of previous studies. IL-2 expression and T cell proliferation are important characteristics of T cell activation. Previous studies have shown that activation of the PD-1/PD-L1 pathway can inhibit T cell proliferation and IL-2 expression (46). PD-1 pathway blockade could promote T cell proliferation, up-regulate the secretion of IFN-γ and IL-2 of lymphocytes, and restore the immune function of HBV-specific T lymphocytes (47). In this study, we found that blocking PD-1 significantly increased IL-2 production and restored PBL proliferation in CP BVDV-infected mice but did not significantly affect PBL proliferation and IL-2 production in NCP BVDV-infected mice, in consistence with the findings of our previous studies (17, 18).

In summary, the presence of hematological abnormalities, viremia, appearance of viral antigens, and histopathological changes provided evidence that the mice were infected with the standard NADL strain and NY-1 strain. We also confirmed the immunomodulatory role of PD-1 in peripheral blood lymphocytopenia in mouse models. Remarkably, PD-1 blockade did not significantly affect PBL proliferation and IL-2 production in NCP BVDV-infected mice. Our findings provided a scientific basis for exploring the mechanism of immune dysfunction caused by acute BVDV infection.

## Data Availability Statement

The raw data supporting the conclusions of this article will be made available by the authors, without undue reservation.

## Ethics Statement

The animal study was reviewed and approved by Management Committee of the Experimental Animal Center of Heilongjiang Bayi Agricultural University.

## Author Contributions

YaL and ZZhao: data collection, analysis, and interpretation, and article drafting. CW, TB and YaL: data collection, analysis and interpretation. YuL, NC and SS: data analysis and interpretation, and article drafting. YuL and ZZhang: critical revision of the article. CF and SZ: data analysis and interpretation, and critical revision of the article. SZ and JC: conception or design of the work, and critical revision of the article. ZZhu and YZ: conception or design of the work, data analysis and interpretation, article drafting, and critical revision of the article. All authors contributed to the article and approved the submitted version.

## Funding

This study was supported by the National Natural Science Foundation of China (32072896), Heilongjiang Postdoctoral Fund (LBH-Z20083), Natural Science Foundation of Heilongjiang Province (LH2021C072) and Heilongjiang Bayi Agricultural University Support Program for San Heng San Zong (TDJH202002 and RRCPY202019).

## Conflict of Interest

The authors declare that the research was conducted in the absence of any commercial or financial relationships that could be construed as a potential conflict of interest.

## Publisher’s Note

All claims expressed in this article are solely those of the authors and do not necessarily represent those of their affiliated organizations, or those of the publisher, the editors and the reviewers. Any product that may be evaluated in this article, or claim that may be made by its manufacturer, is not guaranteed or endorsed by the publisher.
