# Supplementary figures and images for: PD-1 Blockade Restores the Proliferation of Peripheral Blood Lymphocyte and Inhibits Lymphocyte Apoptosis in a BALB/c Mouse Model of CP BVDV Acute Infection
Source: Front Immunol. 2021 Sep 6;12:727254. doi: 10.3389/fimmu.2021.727254 (PMC8450576; doi:10.3389/fimmu.2021.727254)

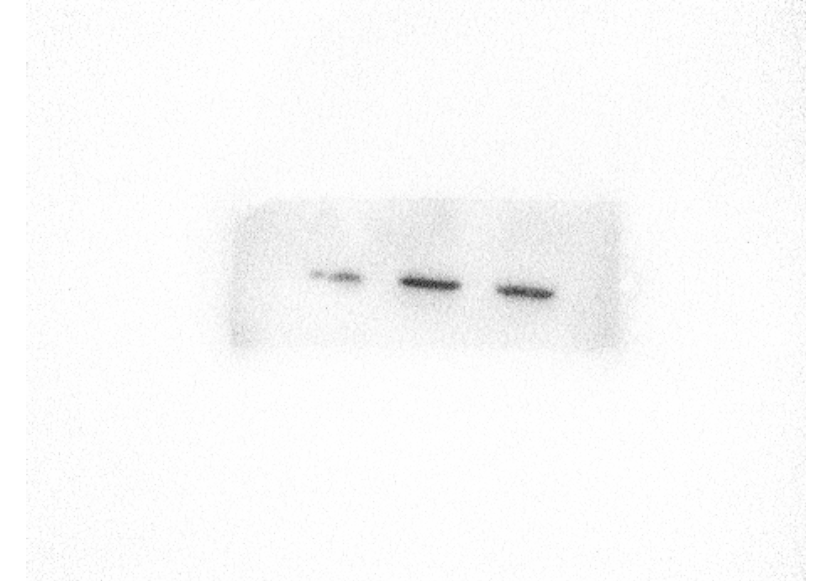

Supplement: Supplementary file 1 [file DataSheet_1.zip › Supplementary material/Supplementary material/PD-1-1.tif]

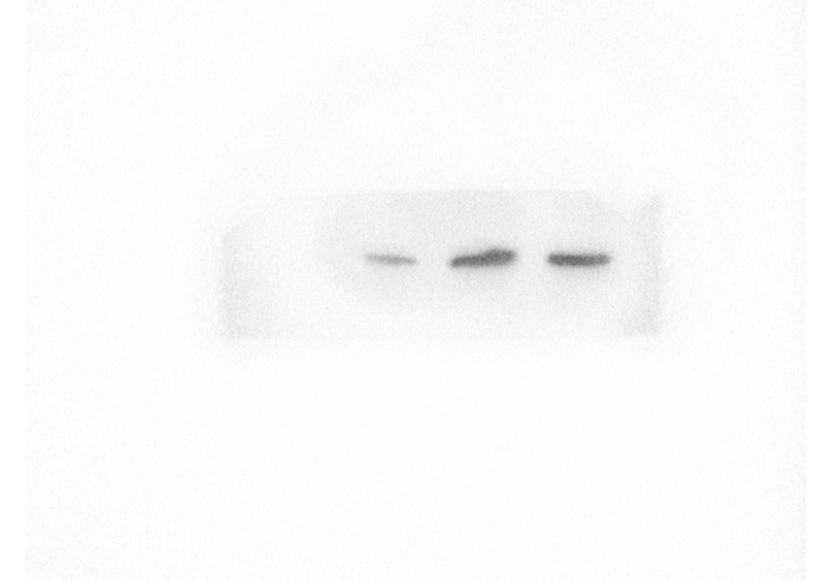

Supplement: Supplementary file 1 [file DataSheet_1.zip › Supplementary material/Supplementary material/PD-1-2.tif]

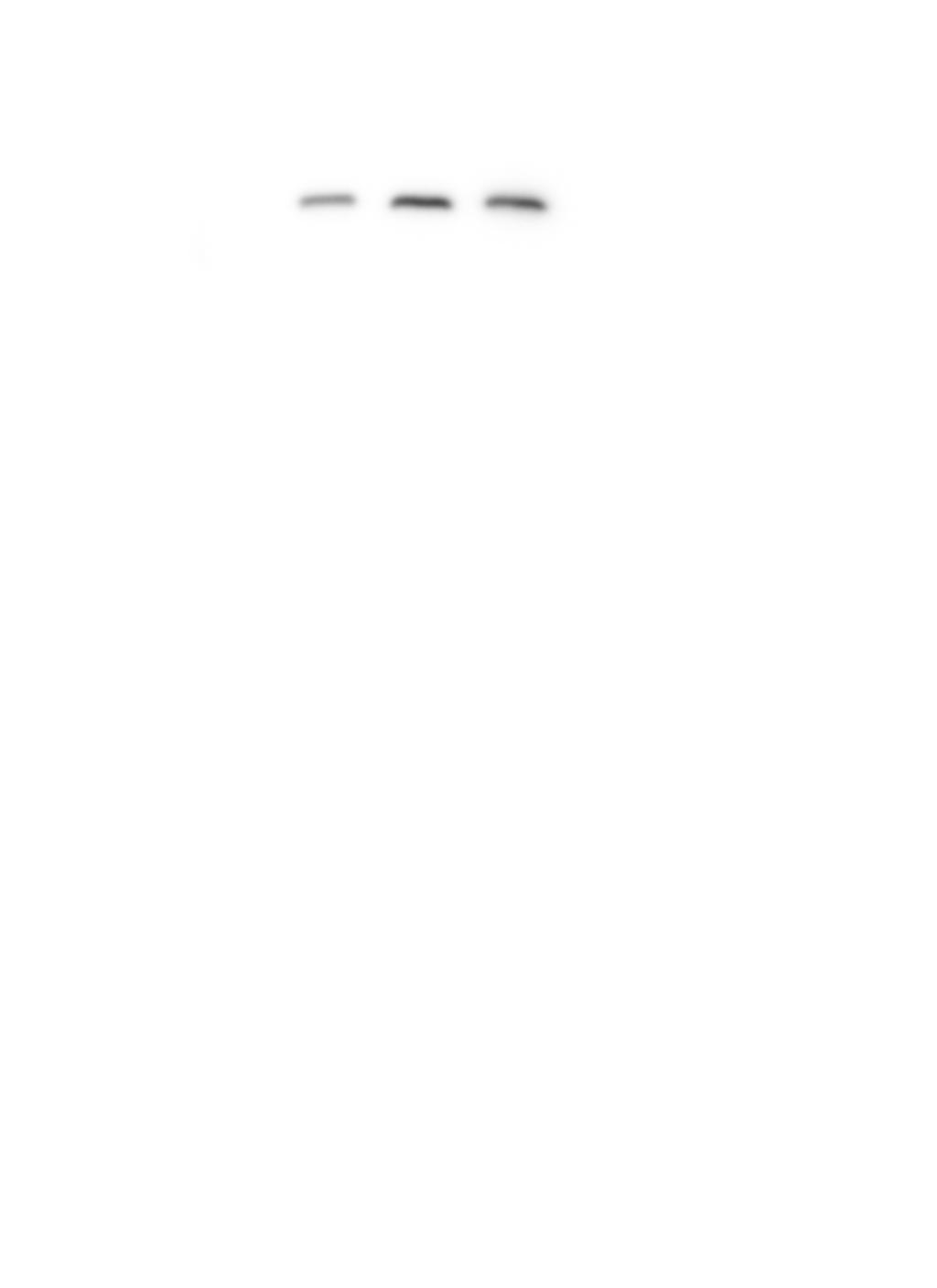

Supplement: Supplementary file 1 [file DataSheet_1.zip › Supplementary material/Supplementary material/PD-1-3.jpg]

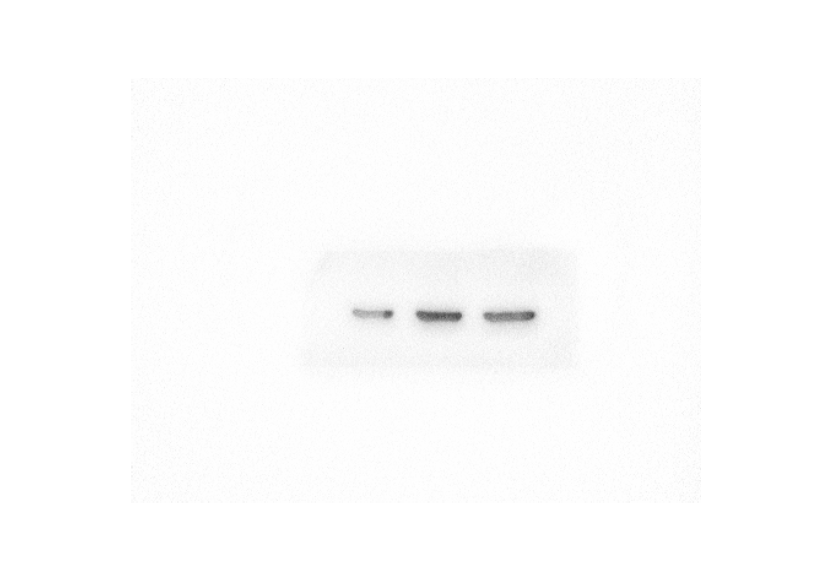

Supplement: Supplementary file 1 [file DataSheet_1.zip › Supplementary material/Supplementary material/PD-1-4.tif]

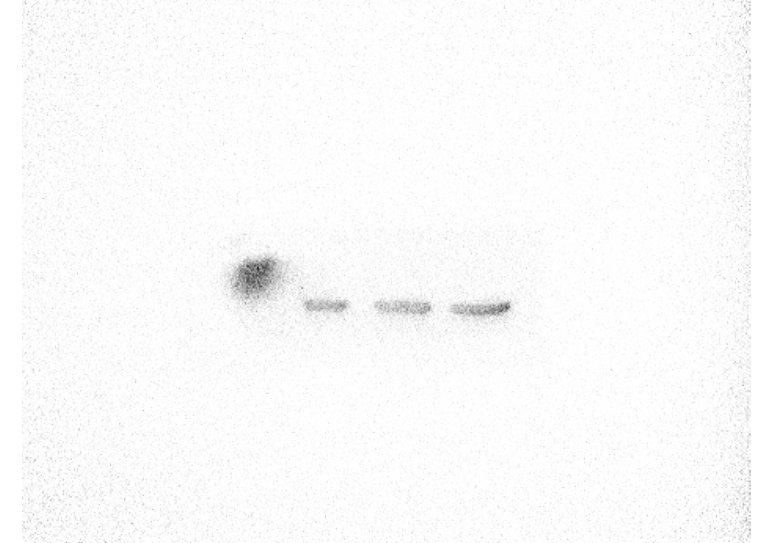

Supplement: Supplementary file 1 [file DataSheet_1.zip › Supplementary material/Supplementary material/PD-L1-1.tif]

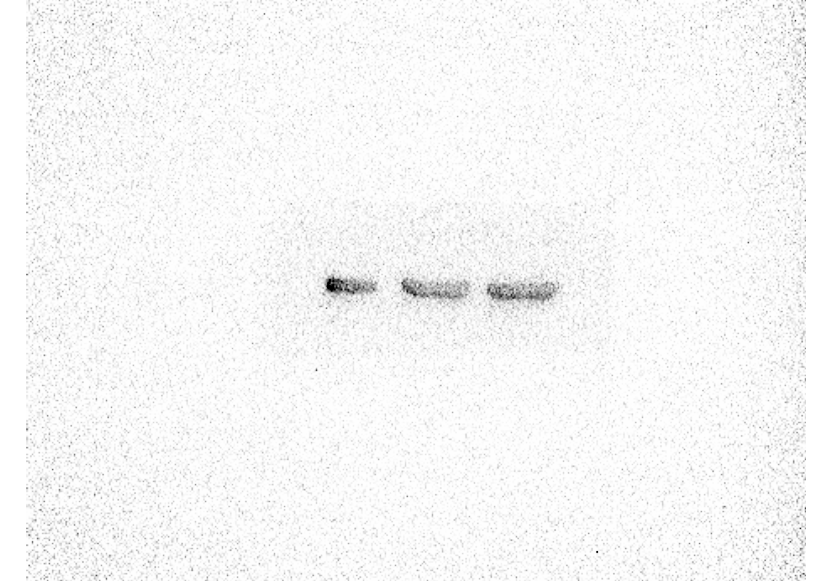

Supplement: Supplementary file 1 [file DataSheet_1.zip › Supplementary material/Supplementary material/PD-L1-2.tif]

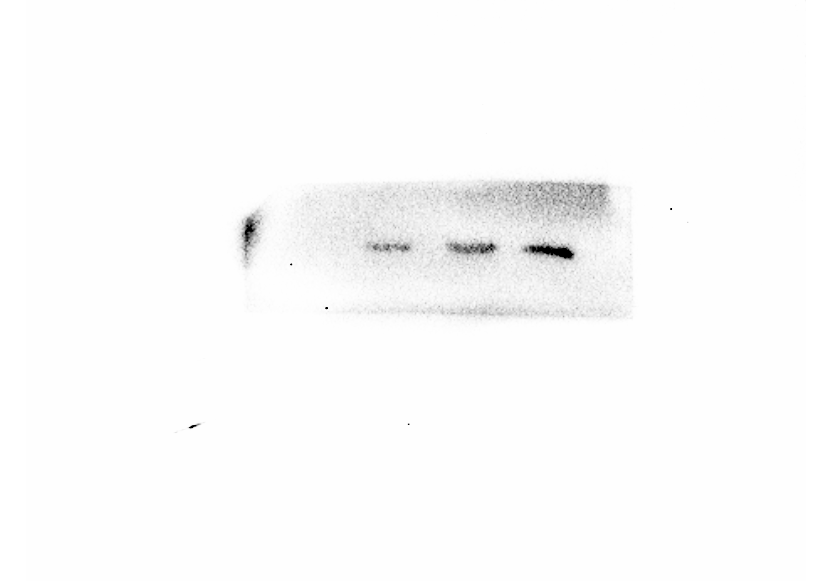

Supplement: Supplementary file 1 [file DataSheet_1.zip › Supplementary material/Supplementary material/PD-L1-3.tif]

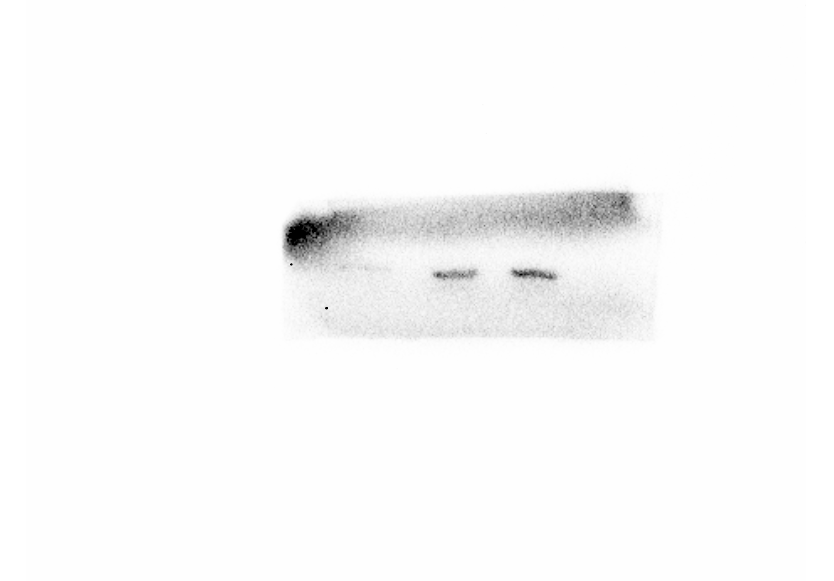

Supplement: Supplementary file 1 [file DataSheet_1.zip › Supplementary material/Supplementary material/PD-L1-4.tif]

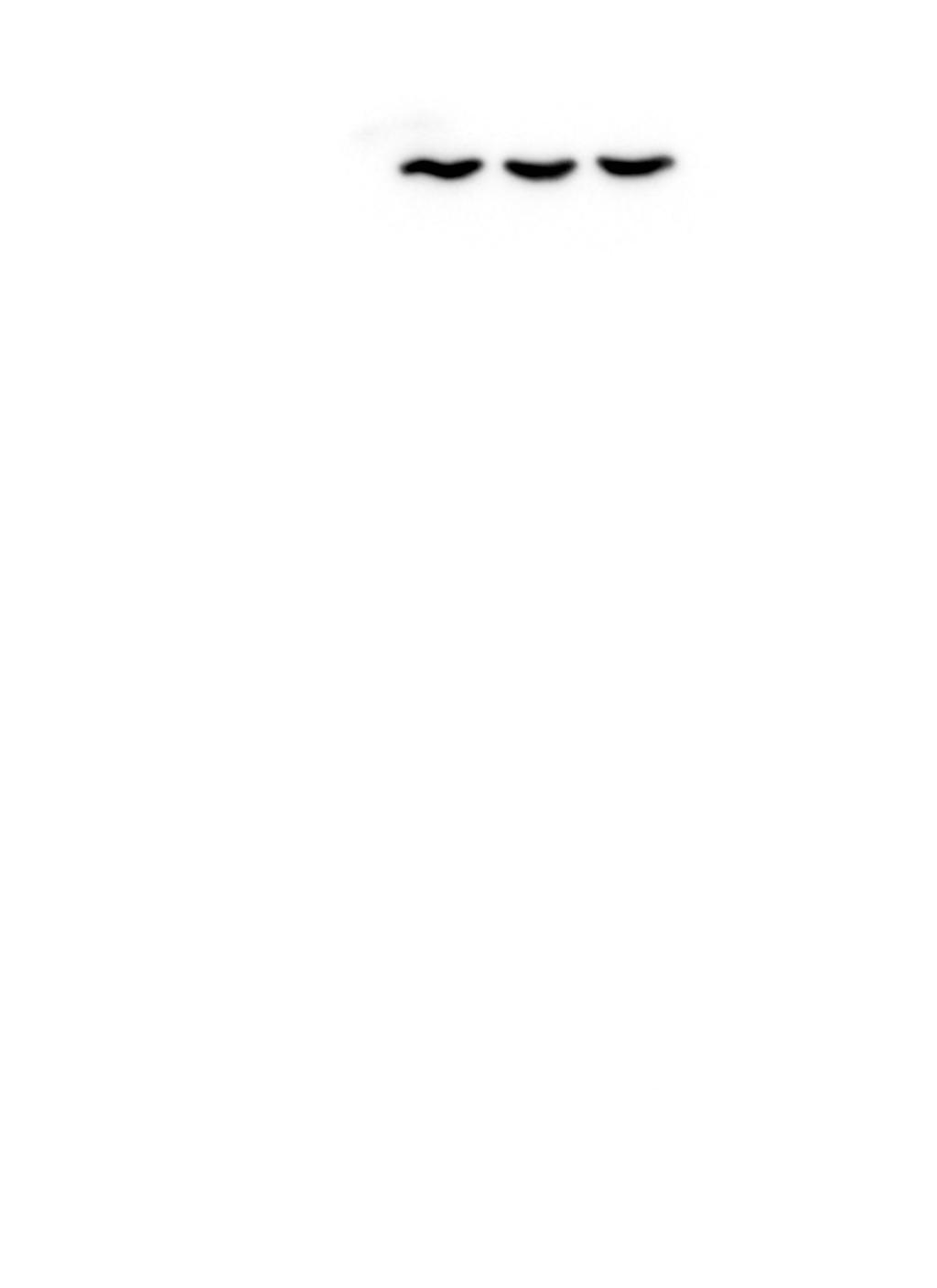

Supplement: Supplementary file 1 [file DataSheet_1.zip › Supplementary material/Supplementary material/β-actin (PD-1)-1.jpg]

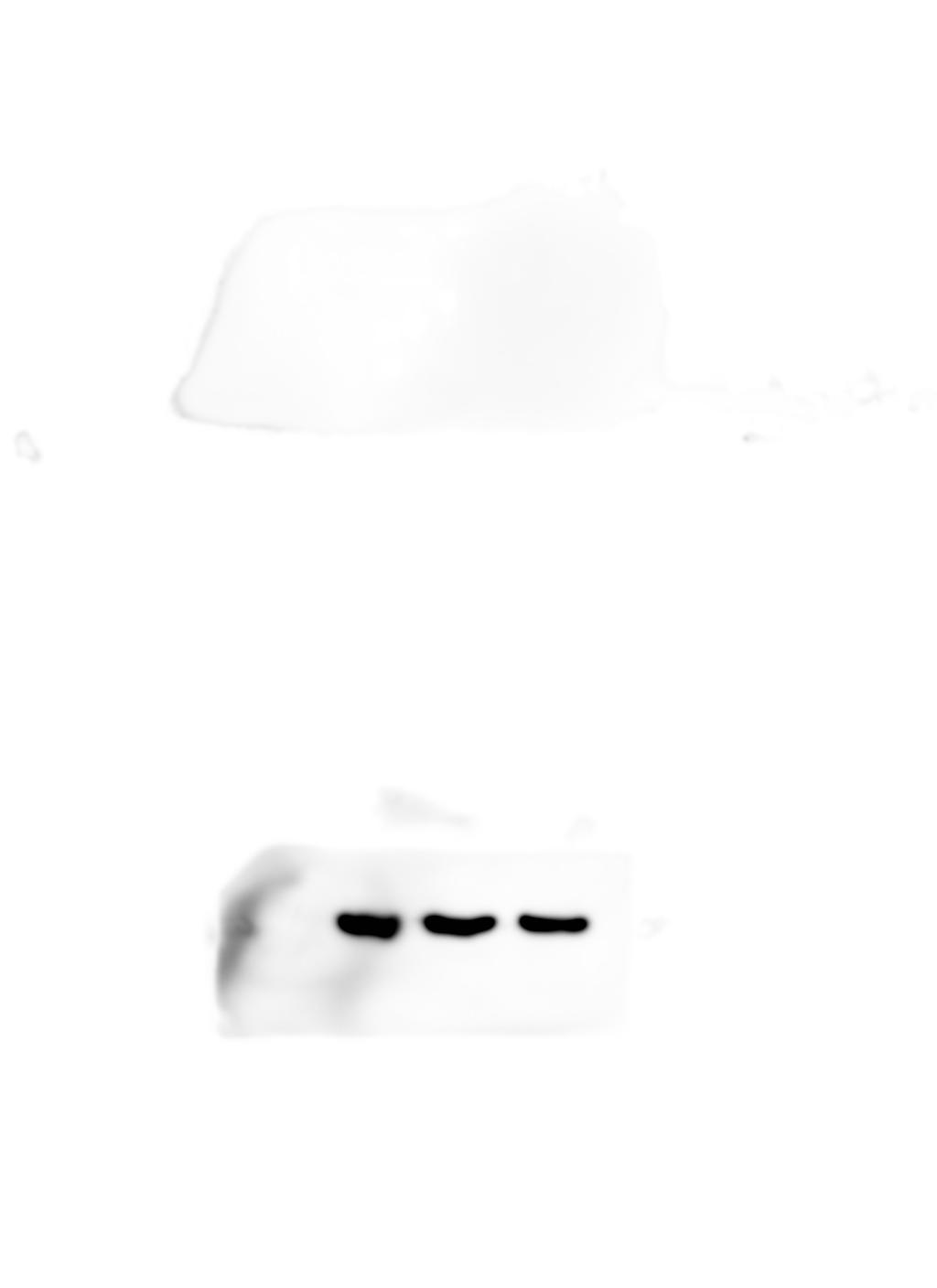

Supplement: Supplementary file 1 [file DataSheet_1.zip › Supplementary material/Supplementary material/β-actin (PD-1)-2.jpg]

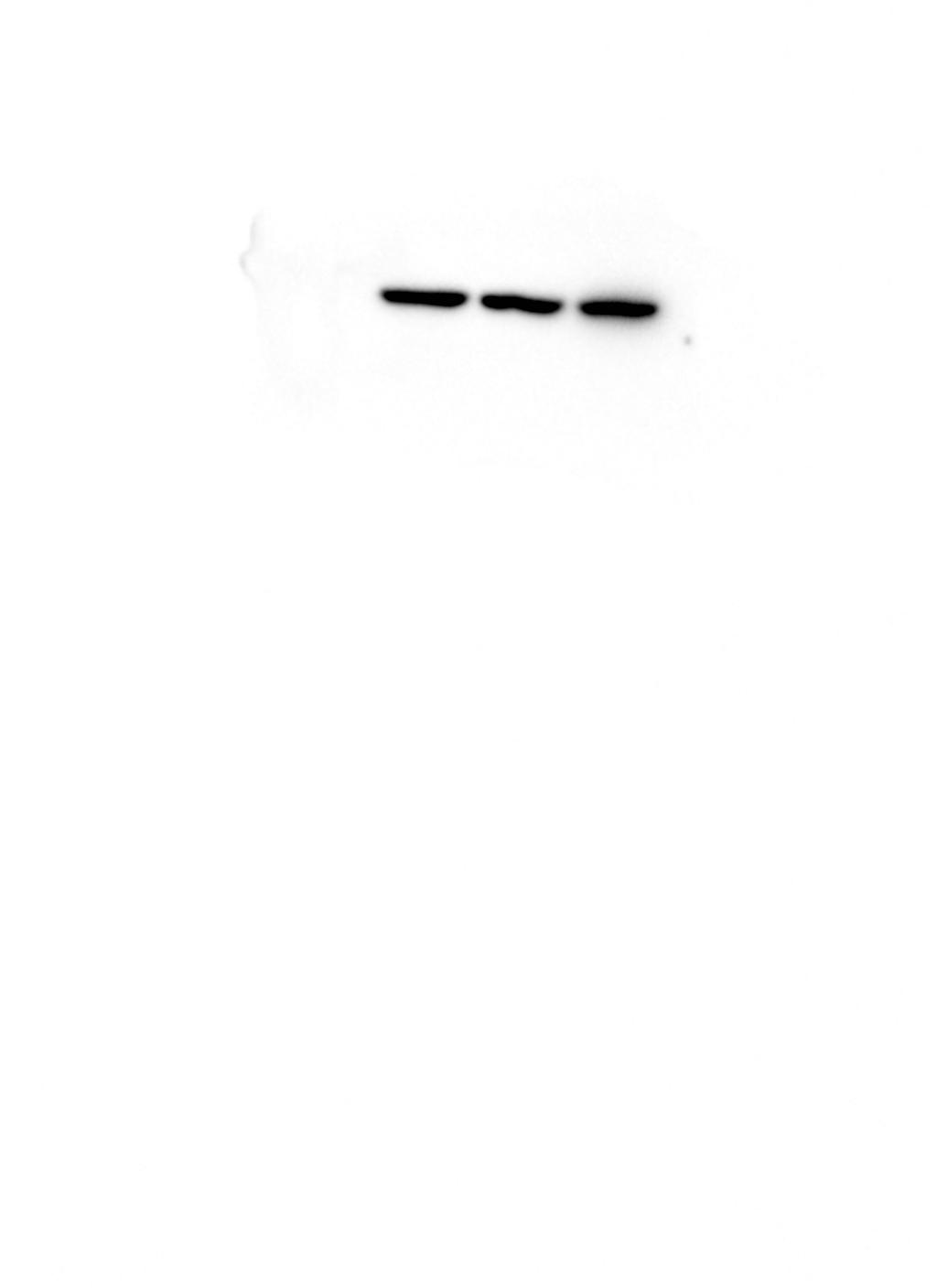

Supplement: Supplementary file 1 [file DataSheet_1.zip › Supplementary material/Supplementary material/β-actin (PD-1)-3.jpg]

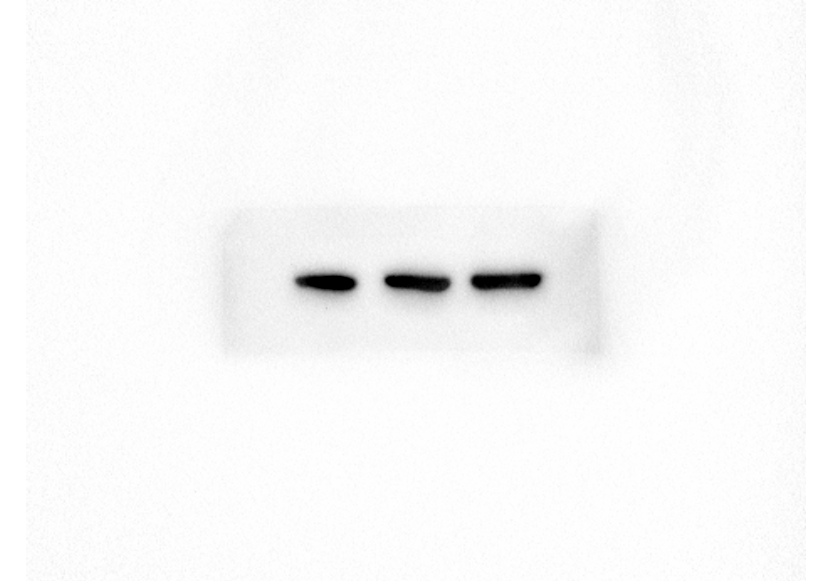

Supplement: Supplementary file 1 [file DataSheet_1.zip › Supplementary material/Supplementary material/β-actin (PD-1)-4.tif]

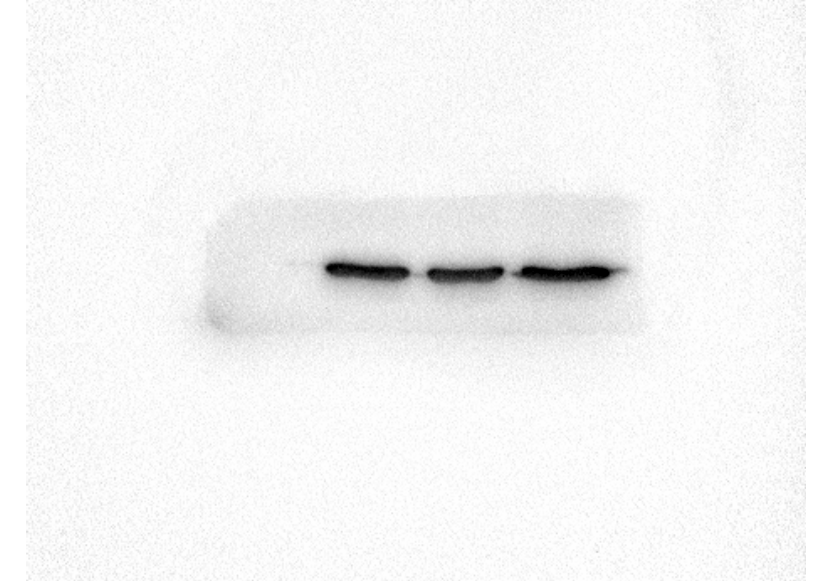

Supplement: Supplementary file 1 [file DataSheet_1.zip › Supplementary material/Supplementary material/β-actin (PD-L1)-1.tif]

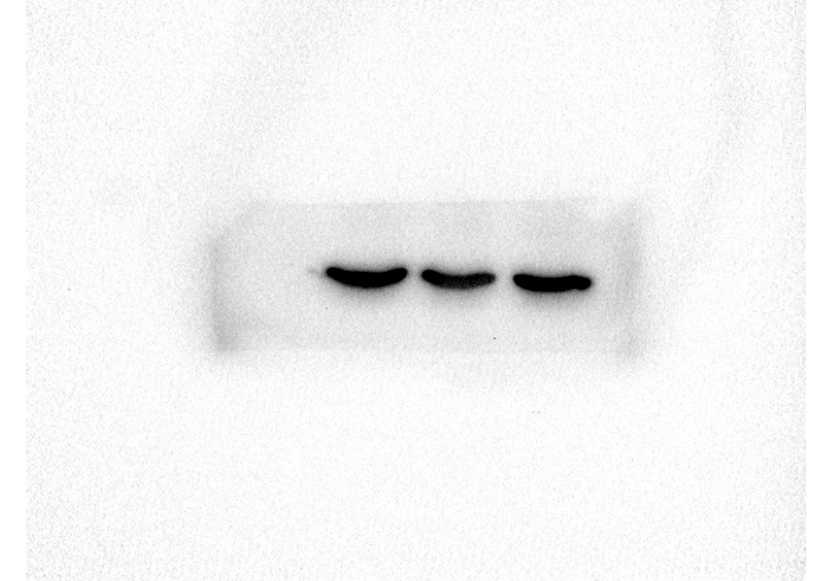

Supplement: Supplementary file 1 [file DataSheet_1.zip › Supplementary material/Supplementary material/β-actin (PD-L1)-2.tif]

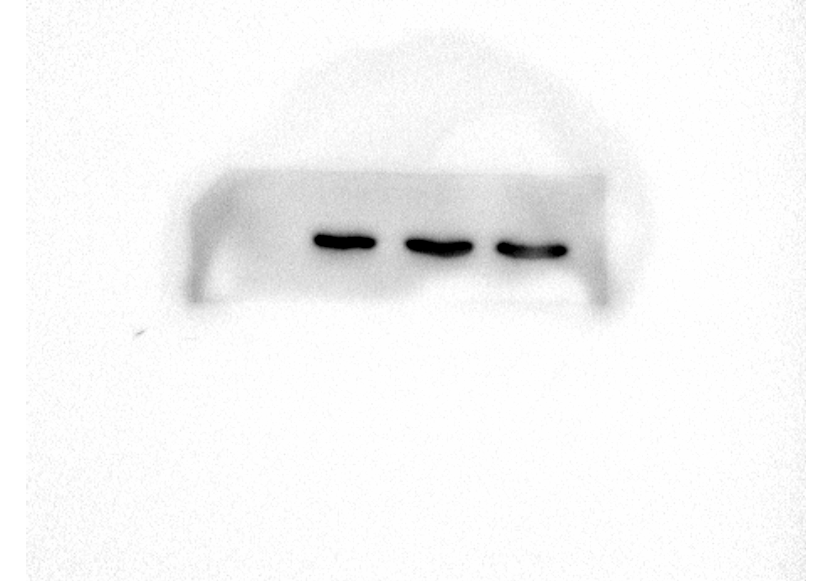

Supplement: Supplementary file 1 [file DataSheet_1.zip › Supplementary material/Supplementary material/β-actin (PD-L1)-3.tif]

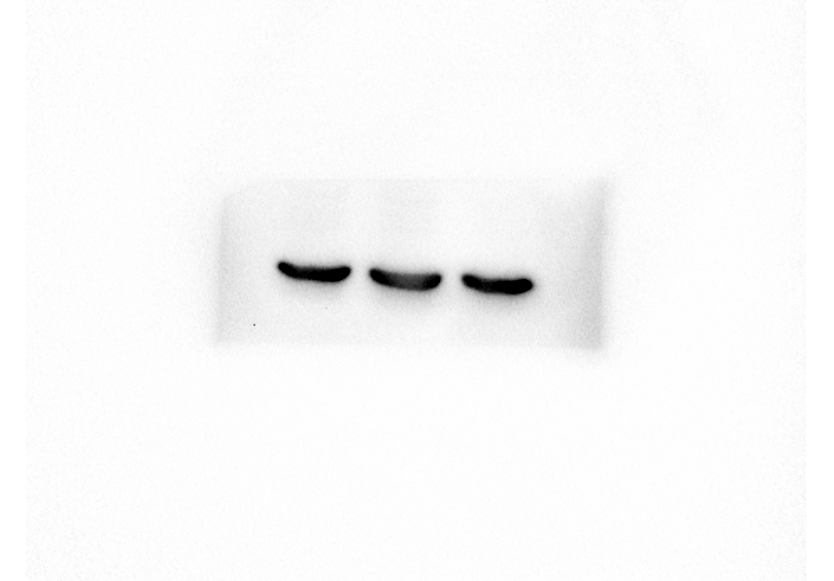

Supplement: Supplementary file 1 [file DataSheet_1.zip › Supplementary material/Supplementary material/β-actin (PD-L1)-4.tif]
